# Comparison of Machine Learning Classifiers for Predicting Reported Antibiotic Treatment After Recent Fever/Cough Among Under‐Five Children: A Pooled Analysis of DHS Data From 64 LMICs

**DOI:** 10.1002/puh2.70413

**Published:** 2026-10-09

**Authors:** Md Sabbir Hossain, Prosenjit Basak Arka, Md Fakrul Islam, Mahfuzer Rohman, Md Jamal Uddin

**Affiliations:** ^1^ Biostatistics, Epidemiology and Public Health Research Team Department of Statistics Shahjalal University of Science and Technology Sylhet Bangladesh; ^2^ Faculty of Graduate Studies Daffodil International University Dhaka Bangladesh

**Keywords:** Demographic and Health Surveys, logistic regression, low‐ and middle‐income countries (LMICs), machine learning, reported antibiotic treatment, under‐five children

## Abstract

**Background:**

Machine learning (ML) methods are increasingly used for health‐related prediction. This study compared logistic regression with five ML algorithms for predicting reported antibiotic treatment following recent fever/cough among children under five using pooled Demographic and Health Survey (DHS) data.

**Methods:**

Data from 64 low‐ and middle‐income countries (LMICs) were analyzed. The final analytic sample included 183,425 children with recent fever/cough, of whom 56,769 (30.95%) were reported to have received antibiotics. Logistic regression, *K*‐nearest neighbors (*K*NN), decision tree, random forest (RF), gradient boosting, and XGBoost were evaluated. The data were divided into model‐development and common heldout test samples, with 55,028 observations in the test set. Hyperparameters were selected using the same three stratified cross‐validation folds for all models, and Synthetic Minority Over‐sampling Technique for Nominal and Continuous (SMOTENC) was applied only within training folds. Model performance was assessed using ROC‐AUC, area under the precision–recall curve (AUPRC), balanced accuracy, sensitivity, specificity, precision, *F*1 score, Matthews correlation coefficient (MCC), Brier score, and calibration measures. Primary classification results used a prespecified probability threshold of 0.50.

**Results:**

Overall predictive performance was modest. RF achieved the highest ROC‐AUC (0.568, 95% CI: 0.552–0.591), whereas *K*NN achieved the highest AUPRC (0.352, 95% CI: 0.308–0.401) and balanced accuracy (0.531, 95% CI: 0.526–0.536). At the 0.50 threshold, sensitivity was low across all models, ranging from 0.105 to 0.252, whereas specificity ranged from 0.810 to 0.910. Calibration was also limited, with calibration slopes ranging from 0.021 to 0.328. No model consistently performed best across all evaluation measures.

**Conclusions:**

ML models did not demonstrate clear overall superiority over logistic regression. Although RF and *K*NN performed better on selected measures, discrimination and calibration remained limited. ML may be useful as a complementary predictive approach, but stronger validation, additional predictors, and more complete consideration of the DHS complex survey design are needed before practical public‐health application.

## Graphical abstract

Using pooled Demographic and Health Survey data from 64 LMICs, six machine learning and logistic regression models showed only modest ability to predict caregiver‐reported antibiotic treatment after childhood fever or cough. No model performed best across all metrics, supporting cautious interpretation and further external validation.

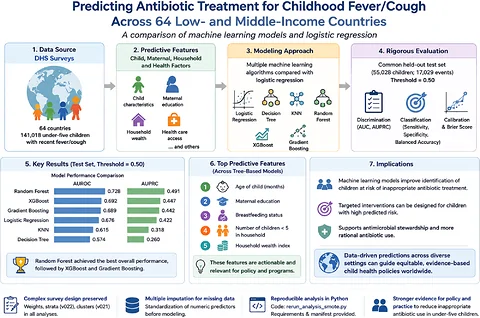

## 1 Introduction

Machine learning (ML) models have played an important role in big data analytic solutions in the past few years [1]. ML techniques have the potential to reduce healthcare expenditures, increase clinical research, and enhance health outcomes [2]. Developing a successful model, deploying it in production, and integrating it with the clinical workflow are difficult tasks for healthcare personnel who frequently lack the requisite ML skills [3]. Modern ML techniques allow researchers to establish conceptual models about how inputs influence results while addressing some of the disadvantages of classic statistical approaches [4]. Supervised learning is widely used in health data prediction, leveraging labeled data to train and test predictive models in public health sectors [5]. Data are typically divided into three subsets: training, validation, and test sets. The training set is utilized to refine the model's parameters for predicting the outcome variables, while the validation set is instrumental in comparing different models and settings [6]. Hyperparameters, predefined before training, significantly influence model performance and require meticulous tuning [6]. Finally, the test set serves to evaluate the final model's capacity to generalize to new data, indicating robustness [6]. Many studies used predictive ML models in health data [7, 8, 9, 10, 11, 12]. For instance, Ren et al. conducted a study on antimicrobial resistance (AMR) prediction using ML. Four ML methods—LR, support vector machine (SVM), RF, and CNN—were applied. Random forest (RF) showed the highest performance with significantly better AUC compared to others. All models effectively predicted AMR with AUC values ranging from 0.69 to 0.96 based on ROC curves [7]. In another study, Yasir et al. proposed an ML method to predict minimum inhibitory concentrations (MICs) of specific antibiotics using genetic data sequences called unitigs. The study found that RF and CATBoost models performed the best in predicting MICs for three antibiotics: cefixime, ciprofloxacin, and azithromycin. The coefficient of determination (*R* ^2^), a measure of how well the regression predictions match the real data points, was 0.76, 0.77, and 0.79 for cefixime, ciprofloxacin, and azithromycin, respectively, using RF. For CATBoost, the *R* ^2^ values were 0.75, 0.77, and 0.79 for cefixime, ciprofloxacin, and azithromycin, respectively [8]. Moreover, Maguire et al. used an ML model on Salmonella samples from broiler chickens. They achieved an average precision between 0.91 and 0.98 by learning about known AMR genes in the samples. The study also pinpointed the main genetic causes of resistance in the Salmonella group, aligning with known AMR mechanisms for specific antibiotics [9]. Furthermore, Her et al. leveraged ML algorithms to forecast the resistance of certain strains to antibiotic drugs, achieving optimal prediction accuracy, particularly for the set of AMR genes located within the accessory part of the pan‐genome. Employing four distinct ML methodologies—SVM, Naive Bayes (NB), RF, and Adaboost—the researchers systematically evaluated their predictive efficacy. Notably, the Adaboost, RF, and SVM algorithms demonstrated marginally superior performance compared to NB, highlighting their potential for discerning patterns indicative of antibiotic resistance in microbial strains [10]. In a study to apply a recently developed ML technique, well‐defined SVM showed comparable performance to logistic regression in cross‐validation for evaluating AMR classification while outperforming RF classifiers significantly. Similarly, in the validation dataset, SVM and logistic regression demonstrated superior performance compared to RF [11]. Ribers et al. employs a two‐step empirical analysis to address the challenge of unnecessary antibiotic prescriptions for urinary tract infections (UTIs). The RF algorithm demonstrates robust performance, evidenced by its ability to accurately predict bacterial risk across patient cohorts, with an observed bacterial UTI rate of 86% among the riskiest 100 patients. Furthermore, the algorithm achieves an area under the receiver operating characteristic curve (AUC) of 0.73, indicating strong predictive capacity comparable to other contexts, such as crime risk assessment [12]. The issue of antibiotic resistance is of global concern and considered a threat to modern healthcare because of misused, inappropriate prescriptions and overuse of antibiotics, which is considered a complex issue with significant implications for public health [13, 14, 15]. Although antibiotic consumption has increased globally, particularly in low‐ and middle‐income countries (LMICs), it has also contributed to a decrease in mortality from antibiotic‐susceptible bacterial infections. The consumption rate of antibiotics increased 78% in LMICs [16]. A study shows that antibiotic drugs are most frequently used by 0–4‐year‐old children [17]. Most of the studies about antibiotic resistance, the datasets are typically small. Complex survey data such as Demographic and Health Survey (DHS) are generally large. Even though antibiotic resistance naturally happens and is a natural result of even the most responsible antibiotic usage, it is obvious that the most recent increases in antibiotic resistance are due to human overuse and abuse of antibiotics [15]. Moreover, antibiotic usage was found to be quite common among children under the age of five in LMICs, and extensive study is required to understand this growing problem. Although antibiotic use among children under five has been examined in several LMICs, previous studies have mainly focused on estimating prevalence, describing prescribing patterns, and identifying factors associated with antibiotic use for common childhood illnesses [18, 19, 20]. Comparatively, little attention has been given to the prediction of reported antibiotic treatment following recent fever or cough using large, pooled, multicountry DHS data. Such prediction is methodologically important because the relationship between socioeconomic, maternal, household, and child characteristics and reported antibiotic treatment may involve nonlinear patterns and interactions that may not be fully captured by conventional logistic regression. At the same time, more flexible ML models do not necessarily provide better prediction than traditional statistical approaches, particularly in heterogeneous survey populations. Therefore, a direct comparison is needed to determine whether ML algorithms provide meaningful predictive improvement over logistic regression in this setting.

The present study was designed as a prediction study rather than a causal or explanatory analysis. Its purpose was not to determine whether particular characteristics cause antibiotic treatment or whether antibiotic use was clinically appropriate. Instead, the study aimed to evaluate and compare the predictive performance of logistic regression and five ML algorithms—*K*‐nearest neighbors (*K*NN), decision tree, RF, gradient boosting, and XGBoost—for predicting reported antibiotic treatment following recent fever/cough among children under five across 64 LMICs, using pooled DHS observations arising from complex survey designs. The study further aimed to assess model discrimination, classification performance, calibration, and predictor importance in order to determine whether more flexible ML methods provide practically meaningful predictive advantages over conventional logistic regression.

## 2 Methods

### 2.1 Data Source and Study Population

This study used data from the DHS conducted in LMICs. The latest eligible standard DHS datasets were initially identified for 92 LMICs from the official DHS Program database. Thirteen countries were excluded because no eligible DHS data were available after 2000, and an additional 15 countries were excluded because of insufficient information or lack of data accessibility, following the criteria used in a previous study [14]. The final analysis, therefore, included data from 64 LMICs.

DHS are nationally representative, population‐based, cross‐sectional household surveys that collect standardized information on population, maternal and child health, nutrition, socioeconomic characteristics, and other health indicators. Standard DHS surveys generally include large household samples and use standardized questionnaires and field procedures, facilitating comparisons across countries.

The initial pooled dataset contained 771,712 children under 5 years of age. Children with recent fever/cough and the information required for the prediction analysis were included in the final analytic sample. The revised analytic sample consisted of 183,425 children, of whom 56,769 (30.95%) were reported to have received antibiotic treatment following recent fever/cough and 126,656 (69.05%) were reported not to have received antibiotic treatment. The sample‐selection procedure is presented in Figure 1, and country‐specific analytic sample sizes and outcome prevalence are reported in Table S5. The DHS datasets are available from the DHS Program after registration, request, and approval.

**FIGURE 1:**
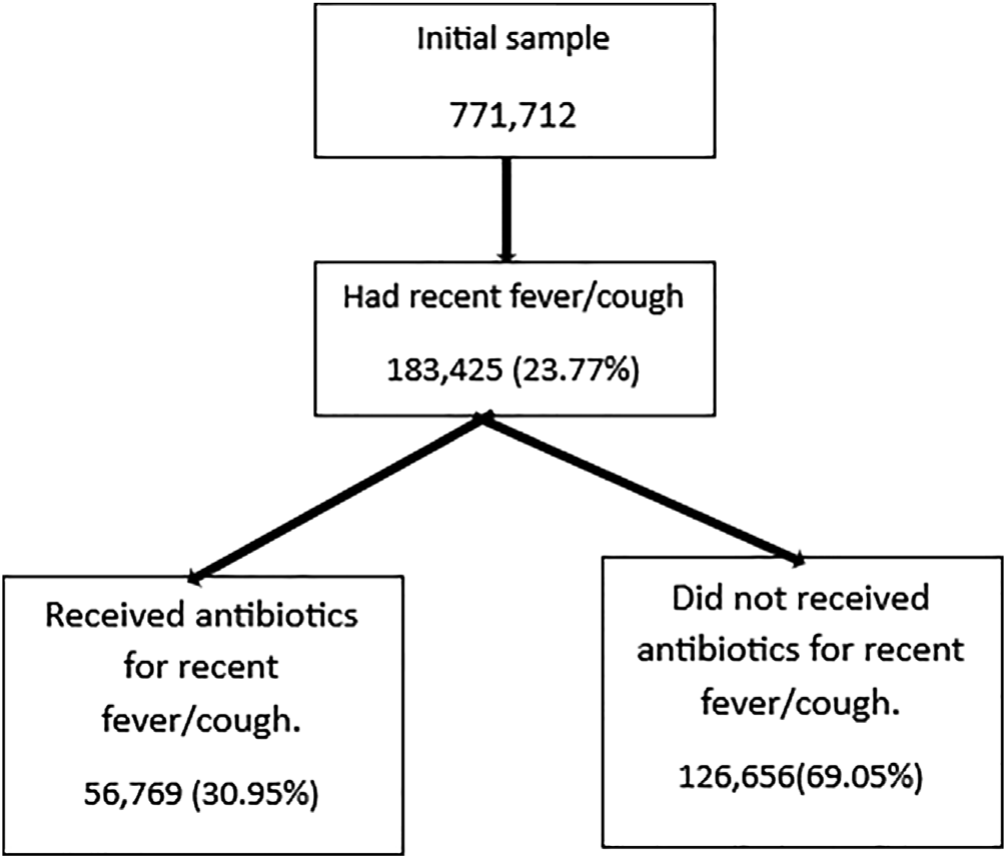
Outcome variables extraction procedures.

### 2.2 Study Design and Handling of the DHS Survey Design

DHS surveys use stratified multistage cluster sampling. In general, sampling strata are formed using geographic regions and urban–rural residence, after which clusters or primary sampling units (PSUs) and households are selected through multiple sampling stages. The surveys are designed to provide nationally and, where applicable, subnationally representative estimates. The present study should be interpreted as a predictive analysis of pooled observations obtained from complex survey datasets rather than a fully design‐based complex‐survey ML analysis. DHS sampling weights were not incorporated directly into model fitting or calculation of the primary predictive‐performance measures. Pooled sampling weights from the 64 surveys were therefore not normalized or rescaled for model development. DHS strata and PSU identifiers were also not explicitly incorporated into model training, train‐test splitting, or Synthetic Minority Over‐sampling Technique for Nominal and Continuous (SMOTENC) resampling. Consequently, countries contributed to model development approximately in proportion to the number of eligible observations available in the pooled analytic dataset, and the reported performance metrics represent unweighted performance within the pooled analytic sample rather than survey‐weighted population‐level performance. To provide uncertainty estimates that reflected the multicountry structure to some extent, 95% confidence intervals for the principal predictive‐performance measures were estimated using a 300‐replicate country‐cluster bootstrap. This procedure does not reproduce all components of the original DHS complex survey design, and this issue is acknowledged as an important limitation of the study.

### 2.3 Data Preprocessing

Initial data cleaning and harmonization were conducted separately for each country before the eligible country datasets were combined. Before any data‐dependent preprocessing, the pooled pre‐imputation analytic dataset was divided into a 70% model‐development sample and a 30% heldout test sample, stratified according to reported antibiotic treatment, using a fixed random seed of 42.

Missing predictor values were handled using multiple imputation by chained equations implemented with the mice package in R. Five imputed datasets were generated using five iterations per imputation. Predictive mean matching was used for numeric and ordinal variables, logistic regression for binary variables, multinomial logistic regression for unordered multicategory variables, and proportional‐odds regression for ordered categorical variables. The imputation model included country and the 12 candidate predictors: place of residence, maternal education, number of children aged 5 years or younger, household wealth index, father's occupation, respondent's occupation, child sex, breastfeeding category, cesarean delivery, vaccination status, child age category, and child BMI category. Country was included only as an auxiliary imputation variable. Case identifiers and the antibiotic‐treatment outcome were excluded from predictor imputation, and observations with missing outcome information were excluded rather than imputed.

Within each of the three cross‐validation folds, the imputation model was fitted exclusively using the fold‐training observations and then applied, without refitting, to the corresponding validation observations. SMOTENC was applied only to the imputed fold‐training data. Categorical encoding and all other learned preprocessing transformations were likewise fitted using the fold‐training data only. Following hyperparameter selection, the imputation procedure was fitted using the complete model‐development sample and applied without refitting to the heldout test predictors. Each classifier was fitted separately across the five imputed datasets, and predicted probabilities were averaged across imputations before calculation of the validation and heldout test performance measures.

### 2.4 Class‐Imbalance Handling

Because reported antibiotic treatment was less frequent than non‐treatment in the analytic sample, class imbalance was addressed during model development using the SMOTENC variables. SMOTENC was applied only within the training portion of each cross‐validation fold. Validation observations and the final heldout test observations were never synthetically resampled.

This procedure ensured that synthetic observations generated to address class imbalance could not enter the validation or test samples, thereby reducing the risk of information leakage and overly optimistic performance estimates.

### 2.5 Outcome Variable

The outcome was reported antibiotic treatment following recent fever/cough among children under 5 years of age. The outcome was coded as a binary variable. Children reported to have received an antibiotic drug or antibiotic injection for their recent fever/cough were coded as 1 (Yes), whereas children who did not receive reported antibiotic treatment for the recent illness were coded as 0 (No). The study predicts reported treatment and does not determine whether antibiotic treatment was clinically indicated, appropriately prescribed, or related to AMR.

### 2.6 Predictor Variables

The 12 predictors represented child, maternal, household, and socioeconomic characteristics available in the DHS datasets. These were place of residence, maternal education, number of children aged 5 years or younger, household wealth index, father's occupation, respondent's occupation, child sex, breastfeeding category, cesarean delivery, vaccination status, child age, and child BMI category. These variables were treated as predictors or predictive features. Their inclusion in the prediction models does not imply that they are causal determinants or independent epidemiological risk factors for antibiotic treatment.

### 2.7 Model Development and Validation

Six classification models were evaluated: logistic regression, *K*NN, decision tree, RF, gradient boosting, and XGBoost. The final analytic dataset of 183,425 observations was divided into a 70% model‐development sample and a 30% heldout test sample. The heldout test set contained 55,028 observations, including 17,029 positive cases. This test set was kept separate from model development and was used only for the final evaluation. Importantly, all six models were evaluated on exactly the same 55,028 heldout observations. Within the model‐development sample, hyperparameter selection was conducted using the same three stratified cross‐validation folds for every model. Mean area under the precision–recall curve (AUPRC) across the three folds was used as the model‐selection criterion. All preprocessing and SMOTENC procedures were refitted independently within each training fold.

The final selected hyperparameters were as follows: logistic regression, *C* = 0.1; *K*NN, *n*_neighbors = 101 with distance weighting; decision tree, max_depth = 12 and min_samples_leaf = 25; gradient boosting, max_iter = 260, learning_rate = 0.05, max_leaf_nodes = 31, and l2_regularization = 2.0; RF, *n*_estimators = 250, max_features = sqrt, and min_samples_leaf = 10; and XGBoost, *n*_estimators = 500, max_depth = 4, learning_rate = 0.05, and min_child_weight = 10. The final hyperparameters and cross‐validated AUPRC values are reported in Table S1, whereas the complete candidate‐model tuning results are provided in Table S10.

A fixed random seed was used to make the splitting, cross‐validation, and model‐fitting procedures reproducible. The exact train‐test and cross‐validation fold assignments were also retained as reproducibility files.

### 2.8 Statistical and ML Techniques

#### 2.8.1 Logistic Regression

Logistic regression is a supervised classification method commonly used for binary outcomes. It models the probability that an observation belongs to the positive outcome class using the logistic function. Predicted probabilities can subsequently be converted to predicted classes using a specified probability threshold [21]. In the present study, logistic regression served as the conventional statistical comparator for the five ML classifiers.

#### 2.8.2 Decision Tree

A decision tree classifies observations through a sequence of decision rules arranged in a tree structure. Internal nodes represent splits on the basis of predictor values, whereas terminal nodes provide the predicted class or class probability. Decision trees are relatively interpretable and can capture nonlinear relationships and interactions among predictors [22].

#### 2.8.3 Random Forest

RF is an ensemble learning method that combines predictions from multiple decision trees. Individual trees are fitted using different bootstrap samples and subsets of candidate predictors, and their predictions are aggregated to obtain the final classification. Combining multiple trees can reduce the variability and overfitting associated with a single decision tree [23].

#### 2.8.4 *K*‐Nearest Neighbors

*K*NN is a nonparametric classification method in which an observation is classified according to the outcomes of observations that are closest to it in the predictor space [24, 25]. The number of neighbors, *K*, influences the bias‐variance trade‐off of the classifier. In the present analysis, *K* was selected through cross‐validation rather than chosen arbitrarily, with the final model using 101 neighbors and distance‐based weighting.

#### 2.8.5 Gradient Boosting

Gradient boosting is an ensemble classification method that sequentially combines weak learners to improve prediction of a binary outcome. Each successive learner focuses on reducing prediction errors from previous iterations. Model complexity and overfitting can be controlled through parameters such as the learning rate, number of boosting iterations, tree complexity, and regularization [26, 27, 28]. In this study, gradient boosting was applied specifically as a binary classification method, rather than as a regression model.

#### 2.8.6 XGBoost

XGBoost is an optimized gradient‐boosted tree algorithm designed for efficient and regularized predictive modeling. It sequentially constructs decision trees while minimizing a specified loss function and controlling model complexity through regularization and other hyperparameters. Parameters such as learning rate, tree depth, number of estimators, and minimum child weight can influence predictive performance and overfitting [29, 30].

### 2.9 Primary Classification Threshold and Sensitivity Analysis

A probability threshold of 0.50 was used for the primary threshold‐dependent classification results for all six models. This common threshold was used to calculate sensitivity, specificity, precision, balanced accuracy, *F*1 score, Matthews correlation coefficient (MCC), and the confusion matrices.

As a secondary sensitivity analysis, an alternative threshold was selected for each model by maximizing the *F*2 score using out‐of‐fold predictions from the model‐development data. Threshold selection was, therefore, performed without using the heldout test outcomes. Results from these *F*2‐selected thresholds were treated as sensitivity analyses and are reported separately in Table S3 rather than being used for the primary conclusions.

### 2.10 Model Performance Measures

Model performance was evaluated using complementary measures of discrimination, classification performance, probability accuracy, and calibration. ROC‐AUC summarized the ability of predicted probabilities to rank positive observations above negative observations across all possible classification thresholds. AUPRC summarized the relationship between precision and sensitivity across thresholds and was emphasized because it is informative when outcome classes are imbalanced.

Sensitivity (recall) is calculated as

$$\mathrm{Sensitivity}=\frac{\mathrm{TP}}{\mathrm{TP}+\mathrm{FN}}$$

and represents the proportion of positive cases correctly identified.

Specificity is calculated as

$$\mathrm{Specificity}=\frac{\mathrm{TN}}{\mathrm{TN}+\mathrm{FP}}$$

and represents the proportion of negative cases correctly identified.

Precision, which is equivalent to positive predictive value (PPV) in this binary classification setting, is calculated as

$$\mathrm{Precision}=\frac{\mathrm{TP}}{\mathrm{TP}+\mathrm{FP}}$$

Precision and PPV were, therefore, not treated as separate performance measures.

Balanced accuracy is calculated as the average of sensitivity and specificity:

$$\mathrm{Balanced}\;\mathrm{accuracy}=\frac{\mathrm{Sensitivity}+\mathrm{Specificity}}{2}$$

and gives equal importance to classification performance in the two outcome groups.

The *F*1 score is calculated as the harmonic mean of precision and sensitivity:

$$\;F1=2\times \frac{\mathrm{Precision}\times \mathrm{Sensitivity}}{\mathrm{Precision}+\mathrm{Sensitivity}}$$

where TP is the true positive, TN is the true negative, FP is the false positive, and FN is the false negative.

The MCC was also calculated because it considers all four components of the confusion matrix and provides a balanced summary of binary classification performance. The Brier score was calculated from the squared difference between the predicted probability and observed binary outcome. Lower Brier scores indicate more accurate probability predictions. Confusion matrices containing true positives (TPs), true negatives (TNs), false positives (FPs), and false negatives (FNs) were generated for every model using the identical heldout test observations. Primary confusion matrices at the 0.50 threshold are reported in Table S2.

### 2.11 Calibration Assessment

Calibration was assessed using the calibration intercept and calibration slope for predicted probabilities on the heldout test sample. Ideal calibration corresponds to an intercept of 0 and a slope of 1. Model‐based 95% Wald confidence intervals were calculated for the calibration intercepts and slopes. Calibration was additionally examined by grouping heldout observations according to predicted probability and comparing the mean predicted probability with the observed event rate. Calibration‐decile results are reported in Table S4, and calibration plots are presented in Figure S2.

### 2.12 Confidence Intervals

For the primary predictive‐performance measures, 95% confidence intervals were estimated using 300‐replicate country‐cluster bootstrap resampling. The same heldout predictions and model definitions were used throughout the bootstrap procedure. Calibration intercepts and slopes were reported with model‐based 95% Wald confidence intervals.

### 2.13 Country‐Specific and Subgroup Analyses

To examine heterogeneity in predictive performance across the pooled datasets, the final models were evaluated separately within the heldout observations from each of the 64 country datasets. Country‐specific performance at the 0.50 classification threshold is reported in Table S6.

Performance was also evaluated within subgroups defined by place of residence, child sex, household wealth, and maternal education. These results are reported in Table S7. The subgroup analyses were descriptive assessments of predictive performance and were not interpreted as evidence of causal effect modification. Because observations from the same countries could contribute to both model‐development and test samples, the country‐specific analyses should be interpreted as within‐country heldout evaluations and not as independent country‐heldout external validation.

### 2.14 Predictor‐Importance Analysis

Predictor importance was evaluated using the heldout XGBoost predictions. Mean absolute SHapley Additive exPlanations (SHAP) values were used to quantify the contribution of individual predictors to the model's predictions. SHAP importance was summarized both at the dummy‐variable level and after aggregation to the original predictor level. The aggregated SHAP results are displayed in Figure 4, and detailed estimates are provided in Table S8. As a complementary robustness analysis, heldout permutation importance was calculated by randomly permuting each predictor and measuring the resulting decrease in AUPRC. This analysis was conducted using a random sample of 12,000 heldout test observations and is reported in Table S9. SHAP and permutation importance measure different aspects of predictive contribution and therefore were not expected to produce identical rankings. Neither method was interpreted as establishing statistical significance, an independent epidemiological association, or a causal relationship.

### 2.15 Ethics Approval, Consent to Participate, and Data Access

The original DHS surveys were conducted according to established ethical procedures, including review by the relevant national and institutional ethics committees and informed consent from survey participants. The present study was a secondary analysis of de‐identified DHS data and involved no new recruitment or direct contact with participants. Permission to access the datasets was obtained from the DHS Program. The study protocol also received approval from the University Grants Commission (UGC) of the People's Republic of Bangladesh. This approval is distinct from the ethical approvals and informed‐consent procedures used by the DHS Program for the original surveys. Participant confidentiality was maintained throughout the secondary analysis.

### 2.16 Software and Reproducibility

The revised modeling pipeline was implemented in Python. The complete executable analysis is retained in Supporting Information. Exact package requirements and software versions are documented in requirements‐analysis.txt. Reproducibility files also include the train‐test split assignments, cross‐validation fold assignments, individual heldout predictions for all six models, ROC and precision–recall curve coordinates, complete hyperparameter‐tuning results, and execution logs.

## 3 Results

### 3.1 Study Population and Descriptive Characteristics

Data from 64 LMICs were included in the analysis. The initial pooled DHS dataset contained 771,712 children under 5 years of age. Of these, 183,425 children met the eligibility criteria based on having recent fever/cough and availability of the variables required for the analysis. Among the eligible children, 56,769 (30.95%) were reported to have received antibiotic treatment for recent fever/cough, whereas 126,656 (69.05%) were reported not to have received antibiotic treatment (Figure 1).

The sex distribution of the initial sample of 771,712 under‐five children was nearly equal, with 51.06% males and 48.94% females (Figure 2). This figure represents the initial under‐five population rather than the final analytic sample.

**FIGURE 2:**
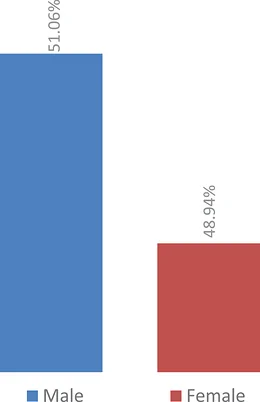
Gender distribution of children under five.

The characteristics of the final analytic sample of 183,425 children are presented in Table 1. Most children lived in rural areas (70.51%), whereas 29.49% lived in urban areas. Regarding maternal education, 30.03% of mothers had no formal education, 31.65% had primary education, 32.04% had secondary education, and 6.28% had higher education. Most households had either one (39.09%) or two (38.78%) children aged 5 years or younger.

**TABLE 1:** Descriptive characteristics of the analytic sample (*N* = 183,425).

Variables	Category	*N* (%)
Type of place of residence	Urban	54,098 (29.49)
	Rural	129,327 (70.51)
Mother's highest educational level	No education	55,074 (30.03)
	Primary	58,045 (31.65)
	Secondary	58,778 (32.04)
	Higher	11,528 (6.28)
Number of children 5 and under	One	71,692 (39.09)
	Two	71,132 (38.78)
	Three	26,660 (14.53)
	Four	7750 (4.23)
	Five or above	6191 (3.38)
Wealth index	Poorest	50,064 (27.29)
	Poorer	41,100 (22.41)
	Middle	35,977 (19.61)
	Richer	31,777 (17.32)
	Richest	24,507 (13.36)
Father's occupation	Did not work	5521 (3.01)
	Agriculture	65,963 (35.96)
	Manual labor	48,957 (26.69)
	Professional/Technical/Managerial	19,211 (10.47)
	Others	43,773 (23.86)
Respondent's occupation	Did not work	85,500 (46.61)
	Agriculture	45,491 (24.80)
	Manual labor	11,359 (6.19)
	Professional/Technical/Managerial	6638 (3.62)
	Others	34,437 (18.77)
Sex of child	Male	95,030 (51.81)
	Female	88,395 (48.19)
Years/Status of breastfeeding	One year	50,420 (27.49)
	Two years	39,063 (21.30)
	Three or more years	14,193 (7.74)
	Ever breastfed, not currently breastfed	73,326 (39.98)
	Never breastfed	6391 (3.48)
	Inconsistent	32 (0.02)
Delivery by cesarean section	No	161,691 (88.15)
	Yes	21,734 (11.85)
Ever had vaccination	No	27,448 (14.96)
	Yes	155,977 (85.04)
Age of child	One year old	43,447 (23.69)
	Two years old	43,556 (23.75)
	Three years old	38,032 (20.73)
	Four years old	32,598 (17.77)
	Five years old	25,792 (14.06)
BMI of the children	Min–9.9	1692 (0.92)
	9.9–14.9	62,696 (34.18)
	14.9–18.5	103,590 (56.48)
	18.5–24.9	13,981 (7.62)
	24.9–max	1466 (0.80)

Approximately 27.29% of children belonged to the poorest wealth category, compared with 13.36% in the richest category. Agriculture was the most common occupation among fathers (35.96%), whereas 46.61% of respondents were not working. Males accounted for 51.81% of the final analytic sample and females for 48.19%.

Regarding breastfeeding history, 39.98% of children had previously been breastfed but were no longer being breastfed, 27.49% were in the 1‐year breastfeeding category, and 21.30% were in the 2‐year category. Most children were delivered without cesarean section (88.15%), and 85.04% had received vaccination. Children were distributed across age categories, with 23.69% aged 1 year, 23.75% aged 2 years, 20.73% aged 3 years, 17.77% aged 4 years, and 14.06% aged 5 years. More than half of the children (56.48%) were in the BMI category of 14.9–18.5 (Table 1).

Sample sizes and outcome prevalence varied substantially across the 64 country datasets. Country‐specific analytic sample sizes, event counts, and outcome prevalence for both the full analytic sample and corresponding heldout test samples are provided in Table S5.

### 3.2 Model Development and Hyperparameter Selection

The final analytic sample was divided into model‐development and heldout test samples. The common heldout test set contained 55,028 observations, including 17,029 children with reported antibiotic treatment, corresponding to an event prevalence of 30.9%. Importantly, all six models were evaluated using exactly the same heldout test observations.

Hyperparameters were selected according to mean AUPRC using the same three stratified cross‐validation folds for all models. The final hyperparameters and mean cross‐validated AUPRC values are reported in Table S1. Mean cross‐validated AUPRC ranged from 0.329 for logistic regression to 0.346 for *K*NN. All preprocessing procedures and SMOTENC resampling were performed within the training folds and were not applied to the validation or heldout test observations. The complete results for the candidate hyperparameter combinations evaluated during model tuning are provided in Table S10.

### 3.3 Predictive Performance on the Common Heldout Test Set

Predictive performance on the identical heldout test sample is summarized in Table 2. Overall, none of the six models demonstrated strong discrimination, and no single model consistently performed best across all performance measures.

**TABLE 2:** Predictive performance of logistic regression and five machine learning models on the common heldout test set.

Model	ROC‐AUC	AUPRC	Balanced accuracy	Sensitivity	Specificity	Precision	*F*1 score	MCC	Brier score
Logistic regression	0.529 (0.511–0.553)	0.329 (0.285–0.385)	0.515 (0.504–0.531)	0.205 (0.174–0.239)	0.825 (0.797–0.854)	0.344 (0.294–0.410)	0.257 (0.229–0.294)	0.036 (0.010–0.074)	0.234 (0.225–0.244)
*K*‐nearest neighbors	0.559 (0.553–0.566)	0.352 (0.308–0.401)	0.531 (0.526–0.536)	0.252 (0.238–0.272)	0.810 (0.796–0.819)	0.373 (0.325–0.424)	0.301 (0.277–0.327)	0.071 (0.058–0.082)	0.260 (0.246–0.276)
Decision tree	0.561 (0.541–0.589)	0.343 (0.295–0.400)	0.508 (0.500–0.519)	0.105 (0.088–0.129)	0.910 (0.892–0.929)	0.344 (0.283–0.427)	0.161 (0.140–0.194)	0.024 (0.001–0.062)	0.224 (0.215–0.235)
Gradient boosting	0.564 (0.546–0.589)	0.344 (0.297–0.400)	0.509 (0.503–0.519)	0.121 (0.104–0.140)	0.898 (0.883–0.914)	0.346 (0.291–0.413)	0.179 (0.159–0.206)	0.028 (0.007–0.057)	0.223 (0.213–0.234)
Random forest	0.568 (0.552–0.591)	0.347 (0.299–0.404)	0.513 (0.505–0.526)	0.144 (0.123–0.173)	0.883 (0.866–0.903)	0.355 (0.300–0.420)	0.205 (0.178–0.238)	0.037 (0.014–0.073)	0.223 (0.213–0.233)
XGBoost	0.562 (0.545–0.586)	0.343 (0.296–0.398)	0.509 (0.503–0.518)	0.124 (0.108–0.145)	0.893 (0.879–0.907)	0.342 (0.293–0.404)	0.182 (0.164–0.209)	0.025 (0.007–0.052)	0.223 (0.213–0.235)

*Note:* Values are estimates with 95% confidence intervals in parentheses. All models were evaluated on the identical heldout test set (*N* = 55,028; events = 17,029). Classification metrics use a threshold of 0.50. ROC‐AUC, AUPRC, and Brier score are threshold‐independent. Higher values indicate better performance except for the Brier score, for which lower values are better. SMOTENC was applied only within training folds and never to validation or heldout test observations.

Abbreviations: AUPRC, area under the precision–recall curve; MCC, Matthews correlation coefficient.

RF had the highest ROC‐AUC, at 0.568 (95% CI: 0.552–0.591). This was followed by gradient boosting at 0.564 (95% CI: 0.546–0.589), XGBoost at 0.562 (95% CI: 0.545–0.586), decision tree at 0.561 (95% CI: 0.541–0.589), *K*NN at 0.559 (95% CI: 0.553–0.566), and logistic regression at 0.529 (95% CI: 0.511–0.553). The ROC curves for all six models are presented in Figure 3 and remained relatively close to the line of no discrimination, consistent with the modest ROC‐AUC values.

**FIGURE 3:**
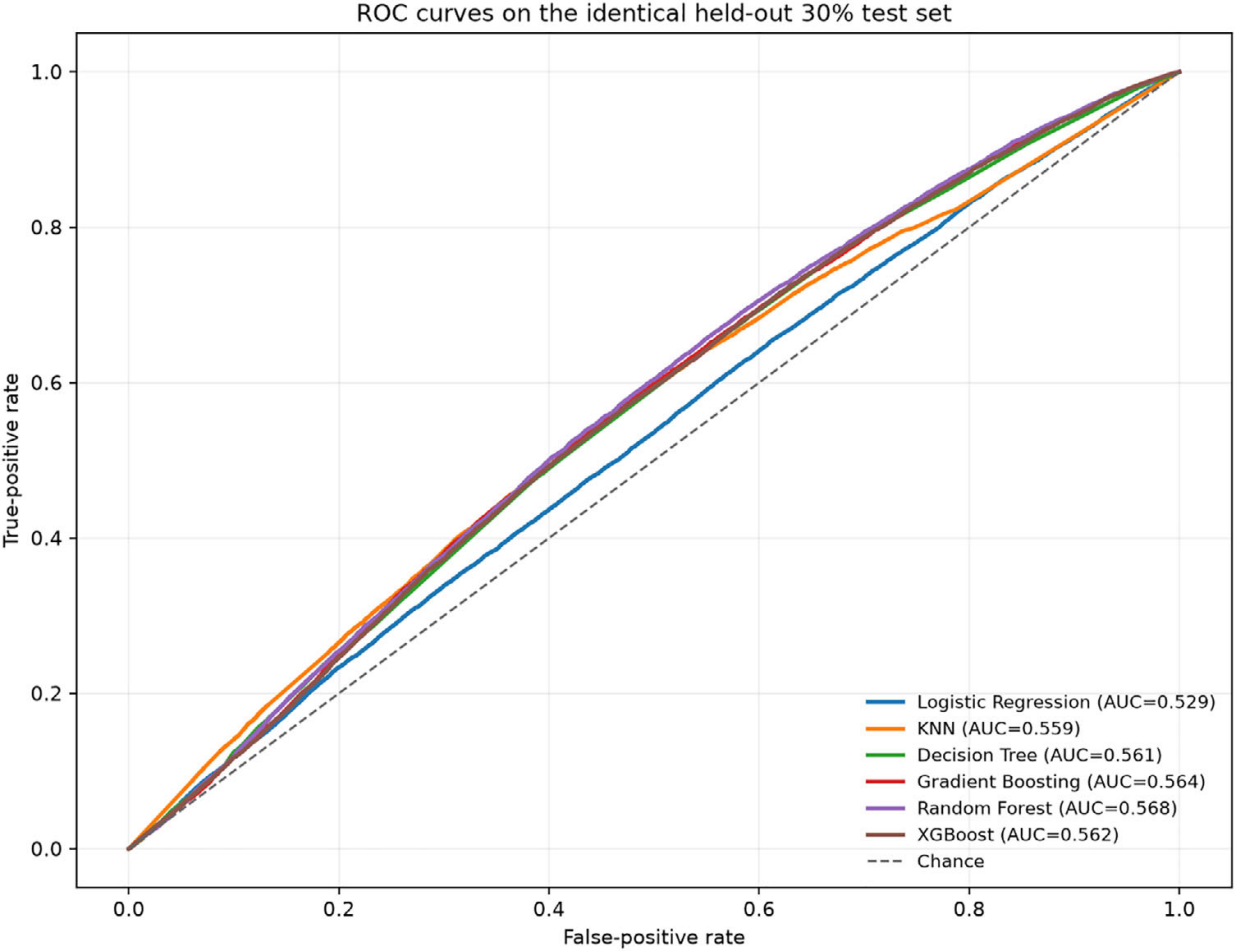
Receiver operating characteristic curves for all models on the identical heldout test set. *K*NN, *K*‐nearest neighbors.

*K*NN achieved the highest AUPRC at 0.352 (95% CI: 0.308–0.401), followed by RF at 0.347 (95% CI: 0.299–0.404), gradient boosting at 0.344 (95% CI: 0.297–0.400), decision tree at 0.343 (95% CI: 0.295–0.400), XGBoost at 0.343 (95% CI: 0.296–0.398), and logistic regression at 0.329 (95% CI: 0.285–0.385). Given the test‐set event prevalence of approximately 0.309, the observed AUPRC values represented only modest improvement in discrimination. The complete precision–recall curves are shown in Figure S1.

At the prespecified probability threshold of 0.50, *K*NN had the highest balanced accuracy, at 0.531 (95% CI: 0.526–0.536), followed by logistic regression at 0.515 (95% CI: 0.504–0.531) and RF at 0.513 (95% CI: 0.505–0.526). Balanced accuracy was 0.508 for decision tree, 0.509 for gradient boosting, and 0.509 for XGBoost.

Sensitivity was low for all models. *K*NN had the highest sensitivity at 0.252 (95% CI: 0.238–0.272), followed by logistic regression at 0.205 (95% CI: 0.174–0.239), RF at 0.144 (95% CI: 0.123–0.173), XGBoost at 0.124 (95% CI: 0.108–0.145), gradient boosting at 0.121 (95% CI: 0.104–0.140), and decision tree at 0.105 (95% CI: 0.088–0.129). Conversely, specificity was relatively high and ranged from 0.810 for *K*NN to 0.910 for decision tree.

*K*NN also had the highest precision (0.373), *F*1 score (0.301), and MCC (0.071). Nevertheless, the relatively low *F*1 scores and MCC values across all models indicate limited ability to correctly distinguish children with and without reported antibiotic treatment. Brier scores ranged from 0.223 to 0.260, with RF, gradient boosting, and XGBoost having similar lowest point estimates of approximately 0.223 (Table 2).

The confusion matrices at the prespecified 0.50 threshold are presented in Table S2. For example, *K*NN correctly classified 4299 of the 17,029 positive cases but failed to identify 12,730 positive cases, whereas decision tree correctly identified 1796 positive cases and missed 15,233. Similar patterns were observed for the other models, demonstrating that the relatively high specificity was accompanied by poor identification of positive cases.

### 3.4 Threshold Sensitivity Analysis

As a sensitivity analysis, classification thresholds were also selected from out‐of‐fold predictions by optimizing the *F*2 score. However, the selected thresholds were extremely low and generated near‐all‐positive classifications. Sensitivity, therefore, approached 1.00, whereas specificity approached zero and balanced accuracy remained approximately 0.50. These results indicated that *F*2 optimization did not provide a practically useful classification rule for this dataset. Consequently, the prespecified threshold of 0.50 was retained for the primary threshold‐dependent analyses. The complete *F*2‐threshold sensitivity analysis is provided in Table S3.

### 3.5 Calibration of Predicted Probabilities

Calibration results are presented in Table 3. None of the six models demonstrated satisfactory calibration. Calibration intercepts ranged from −0.759 for *K*NN to −0.624 for RF, whereas calibration slopes ranged from 0.021 to 0.328, substantially below the ideal calibration slope of 1.

**TABLE 3:** Calibration of predicted probabilities on the common heldout test set.

Model	Calibration intercept	Calibration slope
Logistic regression	−0.733 (−0.756 to −0.711)	0.137 (0.111–0.164)
*K*‐nearest neighbors	−0.759 (−0.778 to −0.740)	0.021 (0.018–0.023)
Decision tree	−0.756 (−0.776 to −0.735)	0.090 (0.071–0.109)
Gradient boosting	−0.646 (−0.671 to −0.621)	0.265 (0.236–0.295)
Random forest	−0.624 (−0.648 to −0.599)	0.328 (0.298–0.359)
XGBoost	−0.642 (−0.667 to −0.617)	0.270 (0.241–0.300)

*Note:* Values are estimates with model‐based 95% Wald confidence intervals. Ideal calibration corresponds to an intercept of 0 and a slope of 1. The slopes substantially below 1 show that the probability predictions were too extreme or did not transport well to the heldout observations. These models should not be used as calibrated probability estimators without additional recalibration and external validation.

RF had the slope closest to the ideal value, at 0.328 (95% CI: 0.298–0.359), followed by XGBoost at 0.270 (95% CI: 0.241–0.300) and gradient boosting at 0.265 (95% CI: 0.236–0.295). Logistic regression had a calibration slope of 0.137 (95% CI: 0.111–0.164), decision tree had a slope of 0.090 (95% CI: 0.071–0.109), and *K*NN had the lowest slope at 0.021 (95% CI: 0.018–0.023) (Table 3).

Observed outcome frequencies and mean predicted probabilities across predicted‐probability groups are reported in Table S4. The corresponding calibration plots are shown in Figure S2. Differences between predicted probabilities and observed event rates became particularly apparent at higher predicted probabilities, indicating that the models should not be interpreted as well‐calibrated probability estimators without further recalibration and external validation.

### 3.6 Country‐Specific and Subgroup Performance

Because data were pooled across 64 countries with differing outcome prevalence and population characteristics, predictive performance was additionally evaluated separately within individual country test samples. Country‐specific performance estimates at the 0.50 threshold are reported in Table S6. These analyses demonstrated heterogeneity in model performance between countries. Because observations from each country were included during model development, these analyses represent within‐country heldout performance rather than country‐heldout external validation.

Performance was also examined across population subgroups defined by place of residence, child sex, household wealth, and maternal education. The subgroup‐specific results are presented in Table S7. These analyses were conducted to assess whether predictive performance varied across important population groups and should be interpreted as descriptive comparisons rather than evidence of causal effect modification.

### 3.7 Predictor Importance

Predictive importance was examined using heldout XGBoost SHAP values. As shown in Figure 4, breastfeeding category had the largest mean absolute SHAP value, followed by child BMI category, cesarean delivery, respondent occupation, father's occupation, maternal education, vaccination status, residence, child age, wealth index, number of children aged 5 years or younger, and child sex. Detailed SHAP results at the original‐predictor and dummy‐variable levels are provided in Table S8.

**FIGURE 4:**
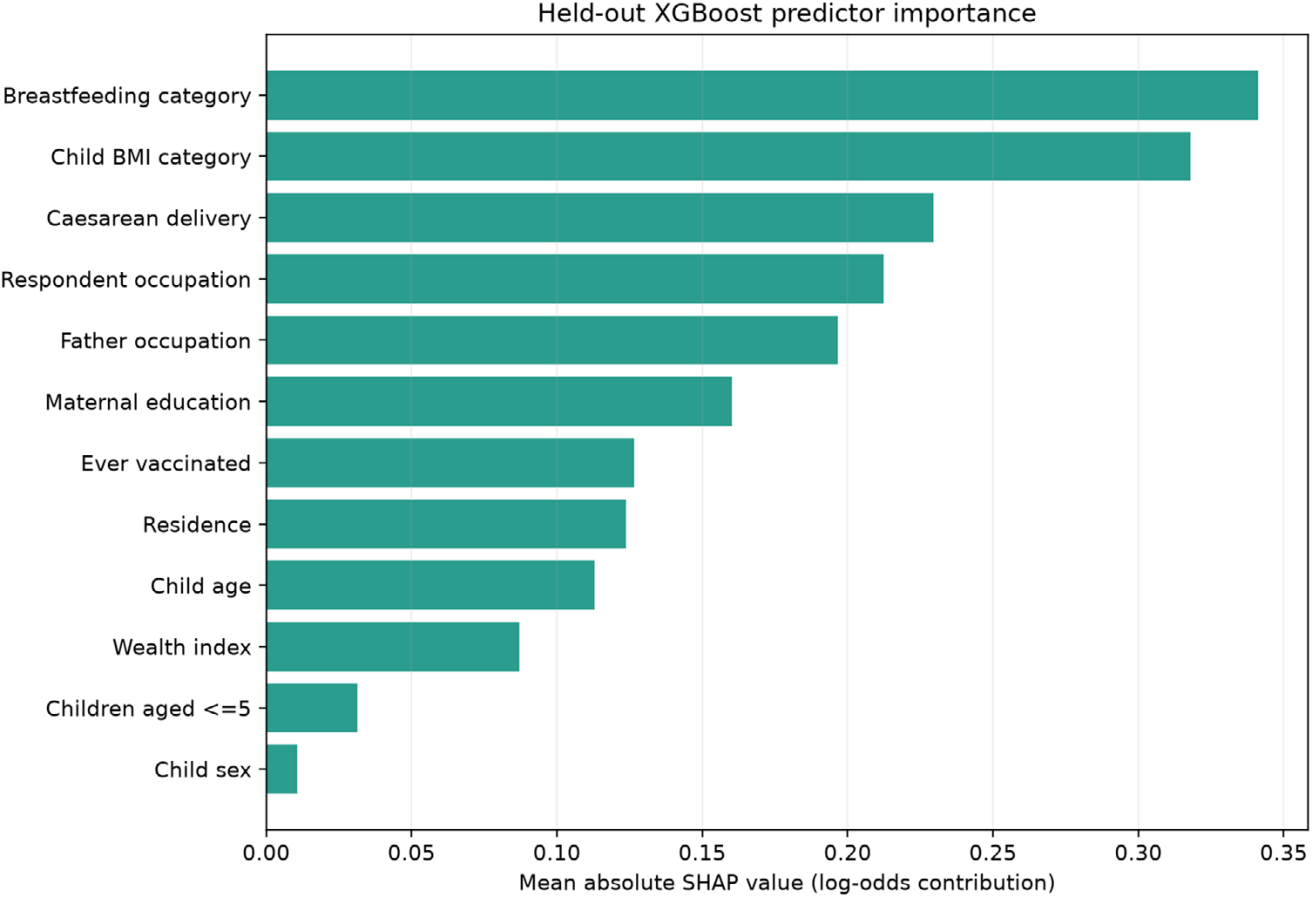
Heldout XGBoost predictor importance based on mean absolute SHAP values. SHAP, SHapley Additive exPlanations.

A complementary permutation‐importance analysis was performed on heldout observations using decrease in AUPRC as the performance measure (Table S9). Child age showed the greatest mean reduction in AUPRC after permutation (0.0177), followed by breastfeeding category (0.0132), maternal education (0.0119), cesarean delivery (0.0081), and residence (0.0054). Respondent occupation, wealth index, child BMI category, and father's occupation produced smaller decreases in AUPRC, whereas child sex contributed little to model performance. Permuting the number of children aged 5 years or younger and vaccination status did not improve predictive importance according to this measure.

The rankings obtained from SHAP and permutation importance were not identical because the two approaches measure different aspects of predictor contribution. These analyses were therefore interpreted only as descriptions of predictive importance. They do not establish statistical significance, independent associations, or causal relationships with reported antibiotic treatment.

## 4 Discussion

This study evaluated the performance of logistic regression and five ML algorithms for predicting reported antibiotic treatment after recent fever/cough among children under 5 years of age using pooled DHS data from 64 LMICs. This study showed that no single model demonstrated consistently superior performance across all evaluation measures. RF had the highest ROC‐AUC (0.568), whereas *K*NN had the highest AUPRC (0.352) and balanced accuracy (0.531). However, the overall discrimination of all models was modest, with ROC‐AUC values ranging from 0.529 to 0.568. These findings suggest that the application of more flexible ML algorithms does not necessarily guarantee substantial improvement over conventional statistical approaches when predicting complex health‐related outcomes from survey‐based socioeconomic and demographic characteristics. This observation is consistent with the broader literature showing that the relative performance of ML and conventional statistical models depends strongly on the outcome, predictor information, data structure, and analytical setting [24, 31, 32, 33].

The results also highlight important trade‐offs between sensitivity and specificity. At the prespecified probability threshold of 0.50, sensitivity remained low across all models, ranging from 0.105 for decision tree to 0.252 for *K*NN, whereas specificity ranged from 0.810 to 0.910. Thus, although several models correctly classified a relatively large proportion of children who did not receive antibiotics, their ability to identify children who received antibiotics was limited. *K*NN produced the highest *F*1 score and MCC among the evaluated models, but these values were also relatively low. Therefore, the performance results should not be interpreted as showing a clear practical advantage of one modeling approach over another. Instead, they demonstrate that different algorithms provide different trade‐offs between identifying positive and negative cases.

The probability‐based performance measures provide further evidence of the limited predictive ability of the models. Although RF achieved the highest ROC‐AUC, the difference between its ROC‐AUC and those of the other ML models was small. Similarly, AUPRC values ranged from 0.329 to 0.352, only modestly above the outcome prevalence of approximately 0.309 in the heldout test sample. Calibration was also poor across all models, with calibration slopes ranging from 0.021 to 0.328, substantially below the ideal value of 1. These findings indicate that the models were limited not only in discriminating between children who did and did not receive reported antibiotic treatment but also in producing well‐calibrated probability estimates. Consequently, these models should not currently be considered suitable for direct clinical or public‐health decision‐making without further model development, recalibration, and external validation.

SMOTENC was applied only within the training folds to avoid information leakage into validation or heldout test observations. In addition, a sensitivity analysis was conducted using thresholds selected by optimizing the *F*2 score from out‐of‐fold predictions. However, the *F*2‐selected thresholds were extremely low and resulted in near‐all‐positive classifications, with sensitivity approaching 1 but specificity approaching zero. This finding demonstrates that improving sensitivity by substantially lowering the classification threshold can produce an unacceptable loss of specificity. Therefore, the prespecified threshold of 0.50 was retained for the primary analysis. These results further emphasize the importance of considering multiple complementary performance measures rather than relying primarily on accuracy when evaluating models for an imbalanced outcome.

Predictor importance was also evaluated using methods that focus on predictive contribution rather than causal interpretation. In the heldout XGBoost SHAP analysis, breastfeeding category showed the greatest mean absolute SHAP value, followed by child BMI category, cesarean delivery, respondent occupation, father's occupation, maternal education, vaccination status, residence, child age, wealth index, number of children aged 5 years or younger, and child sex. A complementary heldout permutation‐importance analysis produced a somewhat different ranking, with child age showing the largest decrease in AUPRC when permuted, followed by breastfeeding category, maternal education, cesarean delivery, and residence. Differences between these rankings are expected because SHAP and permutation importance quantify different aspects of predictive contribution. Importantly, these variables should be described as important predictors or predictive features, rather than significant risk factors or determinants. Previous studies have reported associations between characteristics such as child age, maternal education, breastfeeding, household socioeconomic position, and antibiotic use among children [34, 35]. However, the present predictive analysis does not establish independent associations, statistical significance, direction of effect, or causal relationships.

Previous health‐related ML studies have frequently reported RF as one of the better‐performing classifiers [11, 32, 33, 36]. For example, ML research on AMR has reported favorable performance for RF in some settings [11], whereas a study using Rwanda DHS data identified RF as an effective classifier for predicting infant mortality [36]. Other research has similarly demonstrated the usefulness of RF in health‐related prediction [32, 33]. Although RF achieved the highest ROC‐AUC in the present study, its absolute discrimination remained modest, and it did not perform best according to all other measures. Therefore, our findings provide a more cautious interpretation than simply concluding that RF is universally superior. Differences in the nature of the outcomes, predictor information, study populations, and validation procedures may explain why RF performs strongly in some studies but only modestly in others.

XGBoost also showed performance broadly comparable to several other ML models in the present study, but it did not clearly outperform them. Its ROC‐AUC was 0.562, whereas its AUPRC and balanced accuracy were 0.343 and 0.509, respectively. Previous research has reported strong performance of XGBoost and RF for other health‐prediction problems [37]. However, the current findings demonstrate that the performance of a particular algorithm in one health context cannot necessarily be generalized to another. Predictive performance depends on factors including outcome definition, available features, class distribution, population heterogeneity, preprocessing strategies, and validation design.

Indeed, previous studies have reported considerable variation in which algorithms perform best across different health outcomes. A study predicting the nutritional status of women in Bangladesh reported that SVM performed best across several evaluation measures [38]. In another study predicting diarrhea among under‐five children in Ethiopia, logistic regression outperformed the evaluated ML models [39]. Similarly, research predicting acute respiratory infection among under‐five children in Ethiopia found that an ensemble involving SVM, gradient boosting, and XGBoost performed better than several individual algorithms [40]. Another comparison reported better performance of logistic regression than SVM, *K*NN, and decision tree in a medical prediction setting [41]. Taken together with the present results, these studies suggest that there is no universally superior ML algorithm for health prediction, and model selection should be based on empirical validation for the specific outcome and population rather than on assumptions about the superiority of more complex algorithms.

*K*NN should not be considered one of the poorest performing models because it achieved the highest AUPRC, balanced accuracy, sensitivity, precision, *F*1 score, and MCC at the 0.50 threshold. Nevertheless, its ROC‐AUC remained modest, and it had the highest Brier score among the evaluated models, indicating limitations in its probability predictions. Decision tree, in contrast, demonstrated the highest specificity but the lowest sensitivity, illustrating the trade‐off between correctly identifying negative observations and missing positive cases. Logistic regression also did not consistently outperform the ML algorithms; its ROC‐AUC and AUPRC were the lowest among the six models, although its performance on some threshold‐dependent measures was comparable to several ML approaches. These findings reinforce the importance of evaluating models using several complementary measures rather than ranking algorithms according to a single statistic.

Considerable variation in outcome prevalence and predictive performance was also observed across the 64 countries. Differences between countries in healthcare access, antibiotic availability, treatment practices, socioeconomic conditions, survey periods, and other contextual characteristics may contribute to this heterogeneity. Subgroup analyses by residence, child sex, wealth, and maternal education similarly showed variation in predictive performance. These analyses suggest that pooled performance estimates may conceal important differences between populations. However, the country‐specific analyses represent performance among heldout observations from countries that also contributed data during model development and, therefore, should not be interpreted as independent country‐level external validation. Future studies should evaluate transportability using country‐heldout or external validation designs.

### 4.1 Strength of the Study

A major strength of this study is the use of a large pooled dataset from 64 LMICs to compare logistic regression with five commonly used ML algorithms for predicting reported antibiotic treatment among children under five with recent fever/cough. All six models were evaluated on the same heldout test set, allowing a fair and consistent comparison of their predictive performance. The study also used the same cross‐validation folds for model tuning and applied SMOTENC only within the training folds, thereby reducing the risk of information leakage.

Another important strength is the comprehensive evaluation of model performance. Rather than relying mainly on accuracy, the revised analysis included ROC‐AUC, AUPRC, balanced accuracy, sensitivity, specificity, precision, *F*1 score, MCC, Brier score, and calibration measures, together with 95% confidence intervals. Country‐specific and subgroup‐specific performance analyses were also conducted to examine variation in model performance across populations. In addition, SHAP and permutation‐importance analyses were used to describe the contribution of predictors to model predictions without interpreting them as causal risk factors. These features provide a more transparent and balanced assessment of the usefulness and limitations of ML approaches in pooled DHS data.

### 4.2 Limitations of the Study

This study has several limitations that should be considered when interpreting the findings. First, although the data were obtained from DHS, the ML framework did not fully incorporate all components of the complex survey design. DHS sampling weights were not directly incorporated into model fitting or the calculation of predictive performance measures, and pooled survey weights across the 64 countries were not normalized or rescaled for model development. Similarly, DHS strata and PSU information were not explicitly incorporated into model training, data splitting, or resampling. Therefore, the reported predictive performance measures should be interpreted as model‐based estimates for the pooled analytical sample rather than fully survey‐weighted population‐level estimates. In addition, because countries were pooled without an equal‐country weighting scheme, countries contributing larger analytical samples may have had greater influence on model development and overall performance. Country‐cluster bootstrap procedures were used to quantify uncertainty in the primary performance measures, but this does not fully reproduce all components of the original DHS sampling design.

Second, although all six models were evaluated on the same heldout test set and the same cross‐validation folds were used during model development, the validation procedure was not grouped by country or PSU. Consequently, observations from the same country, and potentially related sampling clusters, could appear in both the model‐development and heldout samples. Although country‐specific and subgroup‐specific performance analyses were conducted, these analyses do not provide the same evidence of transportability as country‐heldout or leave‐one‐country‐out validation. Future studies should consider country‐grouped or PSU‐grouped validation and independent external validation to assess how well the models perform in completely unseen populations.

Third, pooling data from 64 LMICs introduces substantial heterogeneity. Countries differ in healthcare systems, access to antibiotics, treatment practices, socioeconomic conditions, survey periods, and implementation of DHS surveys. Although country‐specific and subgroup performance estimates were examined, the pooled models may not fully capture these differences. The findings should therefore not be interpreted as indicating that model performance will be identical across all LMICs.

Fourth, although class imbalance was addressed using SMOTENC within the training folds and several performance measures appropriate for imbalanced outcomes were evaluated, overall predictive performance remained limited. Discrimination was modest across all models, sensitivity was relatively low at the prespecified probability threshold of 0.50, and calibration slopes were substantially below the ideal value of 1. These findings indicate that the models are not sufficiently accurate or well calibrated for direct clinical or public‐health decision‐making without further model development, recalibration, and external validation.

Fifth, the study was based on cross‐sectional and predominantly caregiver‐reported DHS information. The outcome represents reported antibiotic treatment following recent fever or cough and does not establish whether antibiotic treatment was clinically indicated, appropriately prescribed, or correctly identified by the respondent. Recall error and misclassification may, therefore, be present. Furthermore, because the analysis was designed for prediction rather than causal inference, predictors identified through SHAP or permutation importance should not be interpreted as causal risk factors or determinants of antibiotic treatment.

Finally, the study evaluated logistic regression and five commonly used ML algorithms, but other modeling approaches were not examined. Different algorithms, alternative feature‐engineering strategies, more extensive hyperparameter optimization, and external validation using independent data may result in different predictive performance. Future research should also investigate model fairness, transportability across countries and population groups, and the implications of using predictive algorithms in public‐health settings.

### 4.3 Recommendations

The findings do not support recommending a single ML model as universally superior for predicting reported antibiotic treatment among under‐five children. RF achieved the highest ROC‐AUC, whereas *K*NN performed best according to AUPRC, balanced accuracy, sensitivity, precision, *F*1 score, and MCC at the prespecified threshold of 0.50. However, the differences between models were modest, and overall discrimination, sensitivity, and calibration remained limited.

Therefore, researchers using ML methods with DHS or similar large survey datasets should compare several candidate models rather than selecting an algorithm in advance based on its expected performance. Model evaluation should include measures appropriate for imbalanced outcomes, particularly ROC‐AUC, AUPRC, balanced accuracy, sensitivity, specificity, *F*1 score, MCC, Brier score, and calibration, rather than relying primarily on accuracy.

Future studies should also examine whether model performance can be improved through more informative predictors, alternative modeling approaches, recalibration, and external validation. Validation strategies that separate countries or sampling clusters between model development and evaluation should be considered to provide stronger evidence of transportability. Greater attention should also be given to incorporating survey weights, strata, and PSUs into predictive modeling frameworks when feasible.

Finally, ML models developed from pooled DHS data should not be used directly for clinical or public‐health decision‐making without further validation. Their usefulness should first be confirmed in independent populations, and their calibration, subgroup performance, fairness, and practical consequences should be carefully evaluated.

## 5 Conclusion

In conclusion, this study compared logistic regression and five ML algorithms for predicting reported antibiotic treatment after recent fever/cough among children under five using pooled data from 64 LMICs. The revised analysis showed that no single model consistently outperformed the others across all performance measures. RF achieved the highest ROC‐AUC, while *K*NN showed the highest AUPRC and performed best on several threshold‐dependent measures. Nevertheless, discrimination was modest across all models, sensitivity was generally low, and probability calibration was poor. The predictor‐importance analyses identified breastfeeding category, child BMI, cesarean delivery, respondent and paternal occupation, maternal education, child age, residence, and other characteristics as contributors to model predictions. However, these findings represent predictive importance only and should not be interpreted as statistically significant or causal risk factors. Overall, the results suggest that ML methods can provide a useful complementary approach for analyzing large pooled DHS datasets, but they should not be considered automatically superior to conventional logistic regression. Further work incorporating stronger validation strategies, improved calibration, additional informative predictors, and more complete consideration of the complex survey design is needed before these models can be considered for practical public‐health applications.

## Author Contributions

Prosenjit Basak Arka, Mahfuzer Rohman, Md. Fakrul Islam, and Md. Sabbir Hossain cleaned, compiled, and analyzed the dataset. Prosenjit Basak Arka, Mahfuzer Rohman, Md. Fakrul Islam, and Md. Sabbir Hossain wrote the original draft. Prosenjit Basak Arka, Mahfuzer Rohman, Md. Fakrul Islam, and Md. Sabbir Hossain each contributed equally to this study. Md. Jamal Uddin gave feedback on data analysis and manuscript structure, reviewed and edited the first draft. Md. Jamal Uddin conceptualized, commented, and supervised the study. All authors reviewed the study numerous times. Md. Jamal Uddin has verified the statistical analysis and results of the study and takes full responsibility for the results.

## Funding

This study was funded by the University Grants Commission (UGC) of the People's Republic of Bangladesh, Project ID: Phy‐48‐2021, for the Year 2021–22.

## Consent

This study received publication approval from the University Grants Commission (UGC) of the People's Republic of Bangladesh.

## Conflicts of Interest

The authors declare no conflicts of interest.

## Supporting information




**Table S10**: Complete candidate‐model tuning results using common folds.

**Supporting File**: puh270413‐sup‐0002‐SuppMat.py

**Figure S1**: Precision–recall curves for all models on the heldout test set.
**Figure S2**: Calibration plots by predicted‐probability decile.

**Table S1**: Final hyperparameters and cross‐validated AUPRC for each model.
**Table S2**: Confusion matrices for all models at the prespecified 0.50 threshold.
**Table S3**: Sensitivity analysis using out‐of‐fold *F*2‐selected thresholds.
**Table S4**: Calibration deciles for each model on the heldout test set.
**Table S5**: Sample size and outcome prevalence by country‐wave code.
**Table S9**: Heldout permutation importance measured as decrease in AUPRC.

**Table S6**: Country‐specific predictive performance at the 0.50 threshold.

**Table S7**: Predictive performance across residence, child sex, wealth, and maternal‐education subgroups.

**Table S8**: Heldout XGBoost SHAP importance at original‐predictor and dummy‐variable levels.

## Data Availability

The data that support the findings of this study are openly available in DHS at https://www.dhsprogram.com/data/available‐datasets.cfm.
