# Supplementary material for: Comparison of Machine Learning Classifiers for Predicting Reported Antibiotic Treatment After Recent Fever/Cough Among Under‐Five Children: A Pooled Analysis of DHS Data From 64 LMICs
Source: Public Health Chall. 2026 Oct 9;5(4):e70413. doi: 10.1002/puh2.70413 (PMC13652348; doi:10.1002/puh2.70413)
Supplement: Supplementary file 1 [file PUH2-5-e70413-s004.docx]

**Supplementary Table S1:** Final hyperparameters and cross-validated AUPRC for each model.

| **Model** | **Final hyperparameters** | **Mean CV AUPRC** |
| --- | --- | --- |
| Logistic regression | C=0.1 | 0.329 |
| K-nearest neighbors | n_neighbors=101; weights=distance | 0.346 |
| Decision tree | max_depth=12; min_samples_leaf=25 | 0.339 |
| Gradient boosting | max_iter=260; learning_rate=0.05; max_leaf_nodes=31; l2_regularization=2.0 | 0.342 |
| Random forest | n_estimators=250; max_features=sqrt; min_samples_leaf=10 | 0.344 |
| XGBoost | n_estimators=500; max_depth=4; learning_rate=0.05; min_child_weight=10 | 0.343 |

**Table note:** Hyperparameters were selected by mean AUPRC using the same three stratified cross-validation folds for every model. All preprocessing and SMOTENC resampling were refitted within each training fold.

**Supplementary Table S2:** Confusion matrices for all models at the prespecified 0.50 threshold.

| **Model** | **Threshold** | **N** | **TN** | **FP** | **FN** | **TP** |
| --- | --- | --- | --- | --- | --- | --- |
| Logistic Regression | OOF-selected F2 | 55028 | 4 | 37995 | 1 | 17028 |
| Logistic Regression | 0.5 | 55028 | 31361 | 6638 | 13543 | 3486 |
| KNN | OOF-selected F2 | 55028 | 0 | 37999 | 0 | 17029 |
| KNN | 0.5 | 55028 | 30782 | 7217 | 12730 | 4299 |
| Decision Tree | OOF-selected F2 | 55028 | 0 | 37999 | 0 | 17029 |
| Decision Tree | 0.5 | 55028 | 34575 | 3424 | 15233 | 1796 |
| Gradient Boosting | OOF-selected F2 | 55028 | 15 | 37984 | 1 | 17028 |
| Gradient Boosting | 0.5 | 55028 | 34119 | 3880 | 14976 | 2053 |
| Random Forest | OOF-selected F2 | 55028 | 23 | 37976 | 3 | 17026 |
| Random Forest | 0.5 | 55028 | 33554 | 4445 | 14583 | 2446 |
| XGBoost | OOF-selected F2 | 55028 | 12 | 37987 | 3 | 17026 |
| XGBoost | 0.5 | 55028 | 33917 | 4082 | 14910 | 2119 |

**Supplementary Table S3:** Sensitivity analysis using out-of-fold F2-selected thresholds.

| **Model** | **Threshold** | **Metric** | **Value** | **CI Low** | **CI High** | **CI Method** |
| --- | --- | --- | --- | --- | --- | --- |
| Logistic Regression | OOF-selected F2 | N | 55028 |  |  | 300-replicate country-cluster bootstrap |
| Logistic Regression | OOF-selected F2 | Events | 17029 |  |  | 300-replicate country-cluster bootstrap |
| Logistic Regression | OOF-selected F2 | Prevalence | 0.309461 |  |  | 300-replicate country-cluster bootstrap |
| Logistic Regression | OOF-selected F2 | Accuracy | 0.309515 | 0.268685 | 0.356969 | 300-replicate country-cluster bootstrap |
| Logistic Regression | OOF-selected F2 | Balanced Accuracy | 0.500023 | 0.49994 | 0.500085 | 300-replicate country-cluster bootstrap |
| Logistic Regression | OOF-selected F2 | Sensitivity | 0.999941 | 0.999815 | 1 | 300-replicate country-cluster bootstrap |
| Logistic Regression | OOF-selected F2 | Specificity | 0.000105 | 0 | 0.000193 | 300-replicate country-cluster bootstrap |
| Logistic Regression | OOF-selected F2 | Precision | 0.309471 | 0.268613 | 0.35696 | 300-replicate country-cluster bootstrap |
| Logistic Regression | OOF-selected F2 | NPV | 0.8 | 0.261607 | 1 | 300-replicate country-cluster bootstrap |
| Logistic Regression | OOF-selected F2 | F1 | 0.472659 | 0.423473 | 0.526094 | 300-replicate country-cluster bootstrap |
| Logistic Regression | OOF-selected F2 | MCC | 0.002257 | -0.00664 | 0.007032 | 300-replicate country-cluster bootstrap |
| Logistic Regression | OOF-selected F2 | ROC AUC | 0.52875 | 0.51074 | 0.552777 | 300-replicate country-cluster bootstrap |
| Logistic Regression | OOF-selected F2 | AUPRC | 0.32893 | 0.28494 | 0.384779 | 300-replicate country-cluster bootstrap |
| Logistic Regression | OOF-selected F2 | Brier Score | 0.234272 | 0.224759 | 0.244173 | 300-replicate country-cluster bootstrap |
| Logistic Regression | 0.5 | N | 55028 |  |  | 300-replicate country-cluster bootstrap |
| Logistic Regression | 0.5 | Events | 17029 |  |  | 300-replicate country-cluster bootstrap |
| Logistic Regression | 0.5 | Prevalence | 0.309461 |  |  | 300-replicate country-cluster bootstrap |
| Logistic Regression | 0.5 | Accuracy | 0.633259 | 0.608394 | 0.65851 | 300-replicate country-cluster bootstrap |
| Logistic Regression | 0.5 | Balanced Accuracy | 0.51501 | 0.504232 | 0.531356 | 300-replicate country-cluster bootstrap |
| Logistic Regression | 0.5 | Sensitivity | 0.20471 | 0.174104 | 0.23937 | 300-replicate country-cluster bootstrap |
| Logistic Regression | 0.5 | Specificity | 0.825311 | 0.796995 | 0.853842 | 300-replicate country-cluster bootstrap |
| Logistic Regression | 0.5 | Precision | 0.34433 | 0.293939 | 0.41021 | 300-replicate country-cluster bootstrap |
| Logistic Regression | 0.5 | NPV | 0.698401 | 0.652458 | 0.737141 | 300-replicate country-cluster bootstrap |
| Logistic Regression | 0.5 | F1 | 0.256767 | 0.228583 | 0.29362 | 300-replicate country-cluster bootstrap |
| Logistic Regression | 0.5 | MCC | 0.035817 | 0.009918 | 0.07387 | 300-replicate country-cluster bootstrap |
| Logistic Regression | 0.5 | ROC AUC | 0.52875 | 0.51074 | 0.552777 | 300-replicate country-cluster bootstrap |
| Logistic Regression | 0.5 | AUPRC | 0.32893 | 0.28494 | 0.384779 | 300-replicate country-cluster bootstrap |
| Logistic Regression | 0.5 | Brier Score | 0.234272 | 0.224759 | 0.244173 | 300-replicate country-cluster bootstrap |
| KNN | OOF-selected F2 | N | 55028 |  |  | 300-replicate country-cluster bootstrap |
| KNN | OOF-selected F2 | Events | 17029 |  |  | 300-replicate country-cluster bootstrap |
| KNN | OOF-selected F2 | Prevalence | 0.309461 |  |  | 300-replicate country-cluster bootstrap |
| KNN | OOF-selected F2 | Accuracy | 0.309461 | 0.268592 | 0.356981 | 300-replicate country-cluster bootstrap |
| KNN | OOF-selected F2 | Balanced Accuracy | 0.5 | 0.5 | 0.5 | 300-replicate country-cluster bootstrap |
| KNN | OOF-selected F2 | Sensitivity | 1 | 1 | 1 | 300-replicate country-cluster bootstrap |
| KNN | OOF-selected F2 | Specificity | 0 | 0 | 0 | 300-replicate country-cluster bootstrap |
| KNN | OOF-selected F2 | Precision | 0.309461 | 0.268592 | 0.356981 | 300-replicate country-cluster bootstrap |
| KNN | OOF-selected F2 | NPV |  |  |  | 300-replicate country-cluster bootstrap |
| KNN | OOF-selected F2 | F1 | 0.472654 | 0.423449 | 0.526139 | 300-replicate country-cluster bootstrap |
| KNN | OOF-selected F2 | MCC | 0 | 0 | 0 | 300-replicate country-cluster bootstrap |
| KNN | OOF-selected F2 | ROC AUC | 0.558595 | 0.552593 | 0.565713 | 300-replicate country-cluster bootstrap |
| KNN | OOF-selected F2 | AUPRC | 0.352285 | 0.308422 | 0.400983 | 300-replicate country-cluster bootstrap |
| KNN | OOF-selected F2 | Brier Score | 0.259969 | 0.246442 | 0.276459 | 300-replicate country-cluster bootstrap |
| KNN | 0.5 | N | 55028 |  |  | 300-replicate country-cluster bootstrap |
| KNN | 0.5 | Events | 17029 |  |  | 300-replicate country-cluster bootstrap |
| KNN | 0.5 | Prevalence | 0.309461 |  |  | 300-replicate country-cluster bootstrap |
| KNN | 0.5 | Accuracy | 0.637512 | 0.608687 | 0.661591 | 300-replicate country-cluster bootstrap |
| KNN | 0.5 | Balanced Accuracy | 0.531263 | 0.525555 | 0.536192 | 300-replicate country-cluster bootstrap |
| KNN | 0.5 | Sensitivity | 0.252452 | 0.238136 | 0.272122 | 300-replicate country-cluster bootstrap |
| KNN | 0.5 | Specificity | 0.810074 | 0.796273 | 0.819211 | 300-replicate country-cluster bootstrap |
| KNN | 0.5 | Precision | 0.373307 | 0.325286 | 0.423862 | 300-replicate country-cluster bootstrap |
| KNN | 0.5 | NPV | 0.707437 | 0.66055 | 0.74532 | 300-replicate country-cluster bootstrap |
| KNN | 0.5 | F1 | 0.301209 | 0.276627 | 0.32691 | 300-replicate country-cluster bootstrap |
| KNN | 0.5 | MCC | 0.071053 | 0.058338 | 0.082222 | 300-replicate country-cluster bootstrap |
| KNN | 0.5 | ROC AUC | 0.558595 | 0.552593 | 0.565713 | 300-replicate country-cluster bootstrap |
| KNN | 0.5 | AUPRC | 0.352285 | 0.308422 | 0.400983 | 300-replicate country-cluster bootstrap |
| KNN | 0.5 | Brier Score | 0.259969 | 0.246442 | 0.276459 | 300-replicate country-cluster bootstrap |
| Decision Tree | OOF-selected F2 | N | 55028 |  |  | 300-replicate country-cluster bootstrap |
| Decision Tree | OOF-selected F2 | Events | 17029 |  |  | 300-replicate country-cluster bootstrap |
| Decision Tree | OOF-selected F2 | Prevalence | 0.309461 |  |  | 300-replicate country-cluster bootstrap |
| Decision Tree | OOF-selected F2 | Accuracy | 0.309461 | 0.268592 | 0.356981 | 300-replicate country-cluster bootstrap |
| Decision Tree | OOF-selected F2 | Balanced Accuracy | 0.5 | 0.5 | 0.5 | 300-replicate country-cluster bootstrap |
| Decision Tree | OOF-selected F2 | Sensitivity | 1 | 1 | 1 | 300-replicate country-cluster bootstrap |
| Decision Tree | OOF-selected F2 | Specificity | 0 | 0 | 0 | 300-replicate country-cluster bootstrap |
| Decision Tree | OOF-selected F2 | Precision | 0.309461 | 0.268592 | 0.356981 | 300-replicate country-cluster bootstrap |
| Decision Tree | OOF-selected F2 | NPV |  |  |  | 300-replicate country-cluster bootstrap |
| Decision Tree | OOF-selected F2 | F1 | 0.472654 | 0.423449 | 0.526139 | 300-replicate country-cluster bootstrap |
| Decision Tree | OOF-selected F2 | MCC | 0 | 0 | 0 | 300-replicate country-cluster bootstrap |
| Decision Tree | OOF-selected F2 | ROC AUC | 0.561372 | 0.541204 | 0.589099 | 300-replicate country-cluster bootstrap |
| Decision Tree | OOF-selected F2 | AUPRC | 0.342562 | 0.295343 | 0.399879 | 300-replicate country-cluster bootstrap |
| Decision Tree | OOF-selected F2 | Brier Score | 0.224452 | 0.214774 | 0.23458 | 300-replicate country-cluster bootstrap |
| Decision Tree | 0.5 | N | 55028 |  |  | 300-replicate country-cluster bootstrap |
| Decision Tree | 0.5 | Events | 17029 |  |  | 300-replicate country-cluster bootstrap |
| Decision Tree | 0.5 | Prevalence | 0.309461 |  |  | 300-replicate country-cluster bootstrap |
| Decision Tree | 0.5 | Accuracy | 0.660954 | 0.627139 | 0.689438 | 300-replicate country-cluster bootstrap |
| Decision Tree | 0.5 | Balanced Accuracy | 0.50768 | 0.500213 | 0.519375 | 300-replicate country-cluster bootstrap |
| Decision Tree | 0.5 | Sensitivity | 0.105467 | 0.08751 | 0.128808 | 300-replicate country-cluster bootstrap |
| Decision Tree | 0.5 | Specificity | 0.909892 | 0.892101 | 0.928771 | 300-replicate country-cluster bootstrap |
| Decision Tree | 0.5 | Precision | 0.344061 | 0.282537 | 0.426565 | 300-replicate country-cluster bootstrap |
| Decision Tree | 0.5 | NPV | 0.694166 | 0.648653 | 0.732836 | 300-replicate country-cluster bootstrap |
| Decision Tree | 0.5 | F1 | 0.161445 | 0.13963 | 0.194101 | 300-replicate country-cluster bootstrap |
| Decision Tree | 0.5 | MCC | 0.024231 | 0.000627 | 0.061516 | 300-replicate country-cluster bootstrap |
| Decision Tree | 0.5 | ROC AUC | 0.561372 | 0.541204 | 0.589099 | 300-replicate country-cluster bootstrap |
| Decision Tree | 0.5 | AUPRC | 0.342562 | 0.295343 | 0.399879 | 300-replicate country-cluster bootstrap |
| Decision Tree | 0.5 | Brier Score | 0.224452 | 0.214774 | 0.23458 | 300-replicate country-cluster bootstrap |
| Gradient Boosting | OOF-selected F2 | N | 55028 |  |  | 300-replicate country-cluster bootstrap |
| Gradient Boosting | OOF-selected F2 | Events | 17029 |  |  | 300-replicate country-cluster bootstrap |
| Gradient Boosting | OOF-selected F2 | Prevalence | 0.309461 |  |  | 300-replicate country-cluster bootstrap |
| Gradient Boosting | OOF-selected F2 | Accuracy | 0.309715 | 0.268967 | 0.357161 | 300-replicate country-cluster bootstrap |
| Gradient Boosting | OOF-selected F2 | Balanced Accuracy | 0.500168 | 0.500045 | 0.500379 | 300-replicate country-cluster bootstrap |
| Gradient Boosting | OOF-selected F2 | Sensitivity | 0.999941 | 0.999815 | 1 | 300-replicate country-cluster bootstrap |
| Gradient Boosting | OOF-selected F2 | Specificity | 0.000395 | 0.000162 | 0.000778 | 300-replicate country-cluster bootstrap |
| Gradient Boosting | OOF-selected F2 | Precision | 0.309532 | 0.268689 | 0.357029 | 300-replicate country-cluster bootstrap |
| Gradient Boosting | OOF-selected F2 | NPV | 0.9375 | 0.759135 | 1 | 300-replicate country-cluster bootstrap |
| Gradient Boosting | OOF-selected F2 | F1 | 0.472731 | 0.423567 | 0.526169 | 300-replicate country-cluster bootstrap |
| Gradient Boosting | OOF-selected F2 | MCC | 0.009111 | 0.002834 | 0.015452 | 300-replicate country-cluster bootstrap |
| Gradient Boosting | OOF-selected F2 | ROC AUC | 0.564282 | 0.546231 | 0.588799 | 300-replicate country-cluster bootstrap |
| Gradient Boosting | OOF-selected F2 | AUPRC | 0.343875 | 0.296501 | 0.400277 | 300-replicate country-cluster bootstrap |
| Gradient Boosting | OOF-selected F2 | Brier Score | 0.223111 | 0.212715 | 0.234474 | 300-replicate country-cluster bootstrap |
| Gradient Boosting | 0.5 | N | 55028 |  |  | 300-replicate country-cluster bootstrap |
| Gradient Boosting | 0.5 | Events | 17029 |  |  | 300-replicate country-cluster bootstrap |
| Gradient Boosting | 0.5 | Prevalence | 0.309461 |  |  | 300-replicate country-cluster bootstrap |
| Gradient Boosting | 0.5 | Accuracy | 0.657338 | 0.623049 | 0.686789 | 300-replicate country-cluster bootstrap |
| Gradient Boosting | 0.5 | Balanced Accuracy | 0.509226 | 0.502536 | 0.519378 | 300-replicate country-cluster bootstrap |
| Gradient Boosting | 0.5 | Sensitivity | 0.120559 | 0.104465 | 0.140214 | 300-replicate country-cluster bootstrap |
| Gradient Boosting | 0.5 | Specificity | 0.897892 | 0.883258 | 0.914134 | 300-replicate country-cluster bootstrap |
| Gradient Boosting | 0.5 | Precision | 0.346031 | 0.290803 | 0.41306 | 300-replicate country-cluster bootstrap |
| Gradient Boosting | 0.5 | NPV | 0.694959 | 0.649004 | 0.734226 | 300-replicate country-cluster bootstrap |
| Gradient Boosting | 0.5 | F1 | 0.178817 | 0.159368 | 0.206045 | 300-replicate country-cluster bootstrap |
| Gradient Boosting | 0.5 | MCC | 0.027501 | 0.007325 | 0.057339 | 300-replicate country-cluster bootstrap |
| Gradient Boosting | 0.5 | ROC AUC | 0.564282 | 0.546231 | 0.588799 | 300-replicate country-cluster bootstrap |
| Gradient Boosting | 0.5 | AUPRC | 0.343875 | 0.296501 | 0.400277 | 300-replicate country-cluster bootstrap |
| Gradient Boosting | 0.5 | Brier Score | 0.223111 | 0.212715 | 0.234474 | 300-replicate country-cluster bootstrap |
| Random Forest | OOF-selected F2 | N | 55028 |  |  | 300-replicate country-cluster bootstrap |
| Random Forest | OOF-selected F2 | Events | 17029 |  |  | 300-replicate country-cluster bootstrap |
| Random Forest | OOF-selected F2 | Prevalence | 0.309461 |  |  | 300-replicate country-cluster bootstrap |
| Random Forest | OOF-selected F2 | Accuracy | 0.309824 | 0.269005 | 0.357392 | 300-replicate country-cluster bootstrap |
| Random Forest | OOF-selected F2 | Balanced Accuracy | 0.500215 | 0.500065 | 0.500396 | 300-replicate country-cluster bootstrap |
| Random Forest | OOF-selected F2 | Sensitivity | 0.999824 | 0.999611 | 1 | 300-replicate country-cluster bootstrap |
| Random Forest | OOF-selected F2 | Specificity | 0.000605 | 0.000293 | 0.001031 | 300-replicate country-cluster bootstrap |
| Random Forest | OOF-selected F2 | Precision | 0.309552 | 0.268675 | 0.357102 | 300-replicate country-cluster bootstrap |
| Random Forest | OOF-selected F2 | NPV | 0.884615 | 0.76 | 1 | 300-replicate country-cluster bootstrap |
| Random Forest | OOF-selected F2 | F1 | 0.472741 | 0.423532 | 0.526236 | 300-replicate country-cluster bootstrap |
| Random Forest | OOF-selected F2 | MCC | 0.009128 | 0.003168 | 0.014143 | 300-replicate country-cluster bootstrap |
| Random Forest | OOF-selected F2 | ROC AUC | 0.568424 | 0.552168 | 0.590507 | 300-replicate country-cluster bootstrap |
| Random Forest | OOF-selected F2 | AUPRC | 0.346584 | 0.298666 | 0.403619 | 300-replicate country-cluster bootstrap |
| Random Forest | OOF-selected F2 | Brier Score | 0.222643 | 0.213397 | 0.232694 | 300-replicate country-cluster bootstrap |
| Random Forest | 0.5 | N | 55028 |  |  | 300-replicate country-cluster bootstrap |
| Random Forest | 0.5 | Events | 17029 |  |  | 300-replicate country-cluster bootstrap |
| Random Forest | 0.5 | Prevalence | 0.309461 |  |  | 300-replicate country-cluster bootstrap |
| Random Forest | 0.5 | Accuracy | 0.654212 | 0.621615 | 0.683111 | 300-replicate country-cluster bootstrap |
| Random Forest | 0.5 | Balanced Accuracy | 0.51333 | 0.505135 | 0.526176 | 300-replicate country-cluster bootstrap |
| Random Forest | 0.5 | Sensitivity | 0.143637 | 0.122931 | 0.172599 | 300-replicate country-cluster bootstrap |
| Random Forest | 0.5 | Specificity | 0.883023 | 0.865516 | 0.902784 | 300-replicate country-cluster bootstrap |
| Random Forest | 0.5 | Precision | 0.354956 | 0.299553 | 0.419875 | 300-replicate country-cluster bootstrap |
| Random Forest | 0.5 | NPV | 0.697052 | 0.651016 | 0.735539 | 300-replicate country-cluster bootstrap |
| Random Forest | 0.5 | F1 | 0.204515 | 0.177724 | 0.238344 | 300-replicate country-cluster bootstrap |
| Random Forest | 0.5 | MCC | 0.037237 | 0.014197 | 0.072505 | 300-replicate country-cluster bootstrap |
| Random Forest | 0.5 | ROC AUC | 0.568424 | 0.552168 | 0.590507 | 300-replicate country-cluster bootstrap |
| Random Forest | 0.5 | AUPRC | 0.346584 | 0.298666 | 0.403619 | 300-replicate country-cluster bootstrap |
| Random Forest | 0.5 | Brier Score | 0.222643 | 0.213397 | 0.232694 | 300-replicate country-cluster bootstrap |
| XGBoost | OOF-selected F2 | N | 55028 |  |  | 300-replicate country-cluster bootstrap |
| XGBoost | OOF-selected F2 | Events | 17029 |  |  | 300-replicate country-cluster bootstrap |
| XGBoost | OOF-selected F2 | Prevalence | 0.309461 |  |  | 300-replicate country-cluster bootstrap |
| XGBoost | OOF-selected F2 | Accuracy | 0.309624 | 0.268766 | 0.357138 | 300-replicate country-cluster bootstrap |
| XGBoost | OOF-selected F2 | Balanced Accuracy | 0.50007 | 0.499966 | 0.50024 | 300-replicate country-cluster bootstrap |
| XGBoost | OOF-selected F2 | Sensitivity | 0.999824 | 0.999655 | 1 | 300-replicate country-cluster bootstrap |
| XGBoost | OOF-selected F2 | Specificity | 0.000316 | 0.000154 | 0.000566 | 300-replicate country-cluster bootstrap |
| XGBoost | OOF-selected F2 | Precision | 0.30949 | 0.268614 | 0.357014 | 300-replicate country-cluster bootstrap |
| XGBoost | OOF-selected F2 | NPV | 0.8 | 0.639448 | 1 | 300-replicate country-cluster bootstrap |
| XGBoost | OOF-selected F2 | F1 | 0.472669 | 0.423459 | 0.526144 | 300-replicate country-cluster bootstrap |
| XGBoost | OOF-selected F2 | MCC | 0.00391 | -0.00201 | 0.011998 | 300-replicate country-cluster bootstrap |
| XGBoost | OOF-selected F2 | ROC AUC | 0.562419 | 0.54514 | 0.586465 | 300-replicate country-cluster bootstrap |
| XGBoost | OOF-selected F2 | AUPRC | 0.343045 | 0.295923 | 0.398294 | 300-replicate country-cluster bootstrap |
| XGBoost | OOF-selected F2 | Brier Score | 0.223167 | 0.212684 | 0.234818 | 300-replicate country-cluster bootstrap |
| XGBoost | 0.5 | N | 55028 |  |  | 300-replicate country-cluster bootstrap |
| XGBoost | 0.5 | Events | 17029 |  |  | 300-replicate country-cluster bootstrap |
| XGBoost | 0.5 | Prevalence | 0.309461 |  |  | 300-replicate country-cluster bootstrap |
| XGBoost | 0.5 | Accuracy | 0.654867 | 0.619739 | 0.683847 | 300-replicate country-cluster bootstrap |
| XGBoost | 0.5 | Balanced Accuracy | 0.508505 | 0.502527 | 0.51782 | 300-replicate country-cluster bootstrap |
| XGBoost | 0.5 | Sensitivity | 0.124435 | 0.10827 | 0.144731 | 300-replicate country-cluster bootstrap |
| XGBoost | 0.5 | Specificity | 0.892576 | 0.878542 | 0.90742 | 300-replicate country-cluster bootstrap |
| XGBoost | 0.5 | Precision | 0.341719 | 0.292651 | 0.403694 | 300-replicate country-cluster bootstrap |
| XGBoost | 0.5 | NPV | 0.694636 | 0.648145 | 0.73465 | 300-replicate country-cluster bootstrap |
| XGBoost | 0.5 | F1 | 0.182437 | 0.163795 | 0.208692 | 300-replicate country-cluster bootstrap |
| XGBoost | 0.5 | MCC | 0.024868 | 0.00738 | 0.051543 | 300-replicate country-cluster bootstrap |
| XGBoost | 0.5 | ROC AUC | 0.562419 | 0.54514 | 0.586465 | 300-replicate country-cluster bootstrap |
| XGBoost | 0.5 | AUPRC | 0.343045 | 0.295923 | 0.398294 | 300-replicate country-cluster bootstrap |
| XGBoost | 0.5 | Brier Score | 0.223167 | 0.212684 | 0.234818 | 300-replicate country-cluster bootstrap |
| Logistic Regression | Probability calibration | Calibration Intercept | -0.73322 | -0.75555 | -0.71088 | Model-based 95% Wald interval |
| Logistic Regression | Probability calibration | Calibration Slope | 0.137485 | 0.110824 | 0.164145 | Model-based 95% Wald interval |
| KNN | Probability calibration | Calibration Intercept | -0.75914 | -0.77797 | -0.7403 | Model-based 95% Wald interval |
| KNN | Probability calibration | Calibration Slope | 0.020541 | 0.017881 | 0.0232 | Model-based 95% Wald interval |
| Decision Tree | Probability calibration | Calibration Intercept | -0.75592 | -0.77644 | -0.73539 | Model-based 95% Wald interval |
| Decision Tree | Probability calibration | Calibration Slope | 0.090029 | 0.071162 | 0.108897 | Model-based 95% Wald interval |
| Gradient Boosting | Probability calibration | Calibration Intercept | -0.64591 | -0.67092 | -0.62091 | Model-based 95% Wald interval |
| Gradient Boosting | Probability calibration | Calibration Slope | 0.26544 | 0.235568 | 0.295311 | Model-based 95% Wald interval |
| Random Forest | Probability calibration | Calibration Intercept | -0.62358 | -0.64787 | -0.59928 | Model-based 95% Wald interval |
| Random Forest | Probability calibration | Calibration Slope | 0.328397 | 0.297796 | 0.358997 | Model-based 95% Wald interval |
| XGBoost | Probability calibration | Calibration Intercept | -0.64171 | -0.66671 | -0.61671 | Model-based 95% Wald interval |
| XGBoost | Probability calibration | Calibration Slope | 0.27016 | 0.24053 | 0.29979 | Model-based 95% Wald interval |

**Supplementary Table S4:** Calibration deciles for each model on the held-out test set.

| **Model** | **Bin** | **N** | **Mean Predicted** | **Observed Rate** | **Min Predicted** | **Max Predicted** |
| --- | --- | --- | --- | --- | --- | --- |
| Logistic Regression | 1 | 5527 | 0.215218 | 0.273747 | 0.157991 | 0.23486 |
| Logistic Regression | 2 | 5481 | 0.246854 | 0.279146 | 0.234883 | 0.258438 |
| Logistic Regression | 3 | 5501 | 0.269308 | 0.300309 | 0.25844 | 0.280122 |
| Logistic Regression | 4 | 5506 | 0.294564 | 0.295133 | 0.280122 | 0.310155 |
| Logistic Regression | 5 | 5499 | 0.32432 | 0.321149 | 0.310167 | 0.339825 |
| Logistic Regression | 6 | 5503 | 0.357012 | 0.309104 | 0.339826 | 0.375559 |
| Logistic Regression | 7 | 5502 | 0.397305 | 0.306434 | 0.375569 | 0.423015 |
| Logistic Regression | 8 | 5504 | 0.452935 | 0.322311 | 0.423032 | 0.488181 |
| Logistic Regression | 9 | 5502 | 0.53203 | 0.342603 | 0.488187 | 0.585987 |
| Logistic Regression | 10 | 5503 | 0.70846 | 0.344721 | 0.586005 | 0.987206 |
| KNN | 1 | 16511 | 0.051372 | 0.257828 | 0 | 0.212685 |
| KNN | 2 | 5500 | 0.245395 | 0.278727 | 0.212696 | 0.272578 |
| KNN | 3 | 5503 | 0.299436 | 0.293295 | 0.272606 | 0.326148 |
| KNN | 4 | 5503 | 0.340075 | 0.317463 | 0.326154 | 0.361231 |
| KNN | 5 | 5543 | 0.385328 | 0.337002 | 0.361263 | 0.409091 |
| KNN | 6 | 8289 | 0.467413 | 0.345398 | 0.4091 | 0.5 |
| KNN | 7 | 2676 | 0.561677 | 0.36846 | 0.500009 | 0.64162 |
| KNN | 8 | 5503 | 0.907074 | 0.392695 | 0.642213 | 1 |
| Decision Tree | 1 | 7214 | 0.221949 | 0.223177 | 0.08 | 0.231012 |
| Decision Tree | 2 | 7702 | 0.293139 | 0.25409 | 0.24 | 0.304684 |
| Decision Tree | 3 | 5403 | 0.305067 | 0.301129 | 0.305067 | 0.305067 |
| Decision Tree | 4 | 1872 | 0.328519 | 0.301282 | 0.306122 | 0.336748 |
| Decision Tree | 5 | 8741 | 0.343944 | 0.314953 | 0.34 | 0.343972 |
| Decision Tree | 6 | 8583 | 0.370763 | 0.349412 | 0.344828 | 0.373317 |
| Decision Tree | 7 | 5726 | 0.400887 | 0.345791 | 0.375 | 0.435441 |
| Decision Tree | 8 | 4516 | 0.456889 | 0.381754 | 0.435897 | 0.496075 |
| Decision Tree | 9 | 5271 | 0.664368 | 0.344337 | 0.497041 | 1 |
| Gradient Boosting | 1 | 5510 | 0.202805 | 0.203993 | 0.11317 | 0.230108 |
| Gradient Boosting | 2 | 5513 | 0.252099 | 0.252131 | 0.230108 | 0.268948 |
| Gradient Boosting | 3 | 5495 | 0.278501 | 0.284804 | 0.268956 | 0.287386 |
| Gradient Boosting | 4 | 5493 | 0.298138 | 0.290005 | 0.287389 | 0.308381 |
| Gradient Boosting | 5 | 5505 | 0.320136 | 0.303906 | 0.308414 | 0.33213 |
| Gradient Boosting | 6 | 5511 | 0.345398 | 0.324986 | 0.332133 | 0.358004 |
| Gradient Boosting | 7 | 5495 | 0.370944 | 0.347771 | 0.358029 | 0.384565 |
| Gradient Boosting | 8 | 5500 | 0.402831 | 0.374909 | 0.384566 | 0.424569 |
| Gradient Boosting | 9 | 5503 | 0.460915 | 0.366527 | 0.424579 | 0.509564 |
| Gradient Boosting | 10 | 5503 | 0.647381 | 0.345811 | 0.509592 | 0.998471 |
| Random Forest | 1 | 5503 | 0.19155 | 0.193894 | 0.074083 | 0.228505 |
| Random Forest | 2 | 5504 | 0.250616 | 0.252725 | 0.228515 | 0.269068 |
| Random Forest | 3 | 5506 | 0.285933 | 0.274065 | 0.26908 | 0.301518 |
| Random Forest | 4 | 5500 | 0.315026 | 0.29 | 0.301525 | 0.327575 |
| Random Forest | 5 | 5502 | 0.339553 | 0.314795 | 0.327576 | 0.351233 |
| Random Forest | 6 | 5502 | 0.36343 | 0.332243 | 0.351234 | 0.376224 |
| Random Forest | 7 | 5502 | 0.390972 | 0.352236 | 0.376233 | 0.406915 |
| Random Forest | 8 | 5503 | 0.426547 | 0.35835 | 0.406939 | 0.448512 |
| Random Forest | 9 | 5503 | 0.482071 | 0.380701 | 0.448515 | 0.523294 |
| Random Forest | 10 | 5503 | 0.640619 | 0.34563 | 0.523299 | 0.996616 |
| XGBoost | 1 | 5503 | 0.19523 | 0.204616 | 0.115449 | 0.227329 |
| XGBoost | 2 | 5503 | 0.246748 | 0.255497 | 0.22736 | 0.262924 |
| XGBoost | 3 | 5506 | 0.274313 | 0.274428 | 0.262926 | 0.286037 |
| XGBoost | 4 | 5501 | 0.297283 | 0.300673 | 0.286047 | 0.308167 |
| XGBoost | 5 | 5504 | 0.319607 | 0.30814 | 0.308171 | 0.331558 |
| XGBoost | 6 | 5500 | 0.344496 | 0.323273 | 0.331561 | 0.35821 |
| XGBoost | 7 | 5503 | 0.372394 | 0.347265 | 0.35823 | 0.38819 |
| XGBoost | 8 | 5502 | 0.408846 | 0.376045 | 0.388192 | 0.43274 |
| XGBoost | 9 | 5503 | 0.468689 | 0.361984 | 0.432746 | 0.515308 |
| XGBoost | 10 | 5503 | 0.645245 | 0.342722 | 0.515313 | 0.997674 |

**Supplementary Table S5:** Sample size and outcome prevalence by country-wave code.

| **Country** | **Full_N** | **Full_Events** | **Full_Prevalence** | **Test_N** | **Test_Events** | **Test_Prevalence** |
| --- | --- | --- | --- | --- | --- | --- |
| Afghanistan | 8209 | 3894 | 0.474357 | 2463 | 1168 | 0.474218 |
| Albania | 315 | 155 | 0.492063 | 94 | 46 | 0.489362 |
| Armenia | 177 | 8 | 0.045198 | 53 | 2 | 0.037736 |
| Angola | 2207 | 700 | 0.317173 | 662 | 210 | 0.317221 |
| Azerbaijan | 217 | 99 | 0.456221 | 65 | 30 | 0.461538 |
| Bangladesh | 2988 | 212 | 0.07095 | 897 | 64 | 0.071349 |
| Burkina Faso | 3442 | 1091 | 0.316967 | 1032 | 327 | 0.31686 |
| Benin | 2856 | 447 | 0.156513 | 857 | 134 | 0.156359 |
| Burundi | 5240 | 1072 | 0.20458 | 1572 | 322 | 0.204835 |
| Democratic Republic of the Congo | 7462 | 2313 | 0.309971 | 2239 | 694 | 0.30996 |
| Republic of the Congo | 3209 | 2179 | 0.679028 | 963 | 654 | 0.679128 |
| Côte d’Ivoire | 2167 | 543 | 0.250577 | 650 | 163 | 0.250769 |
| Cameroon | 1581 | 6 | 0.003795 | 475 | 2 | 0.004211 |
| Dominican Republic | 1248 | 501 | 0.401442 | 374 | 150 | 0.40107 |
| Egypt | 4819 | 2703 | 0.560905 | 1446 | 811 | 0.560858 |
| Ethiopia | 1686 | 377 | 0.223606 | 506 | 113 | 0.22332 |
| Gabon | 2377 | 1065 | 0.448044 | 713 | 319 | 0.447405 |
| Ghana | 1217 | 350 | 0.287592 | 365 | 105 | 0.287671 |
| Gambia | 1678 | 645 | 0.384386 | 503 | 193 | 0.383698 |
| Guinea | 1419 | 294 | 0.207188 | 426 | 88 | 0.206573 |
| Guatemala | 4505 | 1742 | 0.386681 | 1352 | 523 | 0.386834 |
| Guyana | 603 | 116 | 0.192371 | 181 | 35 | 0.19337 |
| Honduras | 4424 | 2338 | 0.528481 | 1327 | 701 | 0.528259 |
| Haiti | 2725 | 730 | 0.26789 | 818 | 219 | 0.267726 |
| India | 32486 | 7034 | 0.216524 | 9746 | 2110 | 0.216499 |
| Indonesia | 5709 | 1582 | 0.277106 | 1713 | 475 | 0.277291 |
| Jordan | 1596 | 645 | 0.404135 | 478 | 193 | 0.403766 |
| Kenya | 8386 | 3747 | 0.446816 | 2516 | 1124 | 0.446741 |
| Cambodia | 2061 | 153 | 0.074236 | 618 | 46 | 0.074434 |
| Comoros | 821 | 213 | 0.25944 | 246 | 64 | 0.260163 |
| Kyrgyz Republic | 289 | 148 | 0.512111 | 86 | 44 | 0.511628 |
| Lesotho | 808 | 166 | 0.205446 | 243 | 50 | 0.205761 |
| Morocco | 2192 | 506 | 0.230839 | 658 | 152 | 0.231003 |
| Moldova | 1696 | 702 | 0.413915 | 509 | 211 | 0.414538 |
| Mali | 1685 | 296 | 0.175668 | 506 | 89 | 0.175889 |
| Myanmar | 1288 | 483 | 0.375 | 386 | 145 | 0.375648 |
| Mauritania | 2139 | 118 | 0.055166 | 641 | 35 | 0.054602 |
| Maldives | 682 | 296 | 0.434018 | 205 | 89 | 0.434146 |
| Malawi | 5341 | 1503 | 0.281408 | 1602 | 451 | 0.281523 |
| Mozambique | 1890 | 262 | 0.138624 | 567 | 79 | 0.13933 |
| Nigeria | 982 | 405 | 0.412424 | 294 | 121 | 0.411565 |
| Nicaragua | 832 | 84 | 0.100962 | 249 | 25 | 0.100402 |
| Niger | 2337 | 218 | 0.093282 | 701 | 65 | 0.092725 |
| Namibia | 1569 | 666 | 0.424474 | 471 | 200 | 0.424628 |
| Nepal | 1025 | 343 | 0.334634 | 308 | 103 | 0.334416 |
| Peru | 2136 | 386 | 0.180712 | 641 | 116 | 0.180967 |
| Papua New Guinea | 917 | 511 | 0.557252 | 275 | 153 | 0.556364 |
| Philippines | 700 | 472 | 0.674286 | 210 | 142 | 0.67619 |
| Pakistan | 4742 | 1735 | 0.365879 | 1423 | 521 | 0.366128 |
| Rwanda | 1045 | 702 | 0.67177 | 314 | 211 | 0.671975 |
| Sierra Leone | 1594 | 424 | 0.265997 | 478 | 127 | 0.26569 |
| Senegal | 3136 | 760 | 0.242347 | 941 | 228 | 0.242295 |
| São Tomé and Príncipe | 430 | 174 | 0.404651 | 129 | 52 | 0.403101 |
| Eswatini | 787 | 142 | 0.180432 | 236 | 43 | 0.182203 |
| Chad | 4643 | 971 | 0.209132 | 1393 | 291 | 0.208902 |
| Togo | 2248 | 667 | 0.296708 | 674 | 200 | 0.296736 |
| Tajikistan | 631 | 381 | 0.603803 | 189 | 114 | 0.603175 |
| Timor-Leste | 983 | 245 | 0.249237 | 294 | 73 | 0.248299 |
| Tanzania | 1815 | 330 | 0.181818 | 545 | 99 | 0.181651 |
| Uganda | 5837 | 1814 | 0.310776 | 1751 | 544 | 0.31068 |
| Yemen | 5859 | 2773 | 0.473289 | 1758 | 832 | 0.473265 |
| South Africa | 637 | 201 | 0.315542 | 191 | 60 | 0.314136 |
| Zambia | 1645 | 548 | 0.333131 | 493 | 164 | 0.332657 |
| Zimbabwe | 1055 | 383 | 0.363033 | 317 | 115 | 0.362776 |

**Supplementary Table S6:** Country-specific predictive performance at the 0.50 threshold.

**Supplementary Table S6** can be found as a separate CSV file.

**Supplementary Table S7:** Predictive performance across residence, child sex, wealth, and maternal-education subgroups.

**Supplementary Table S7** can be found as a separate CSV file.

**Supplementary Table S8:** Held-out XGBoost SHAP importance at original-predictor and dummy-variable levels

**Supplementary Table S8** can be found as a separate CSV file.

**Supplementary Table S9:** Held-out permutation importance measured as decrease in AUPRC.

| **Feature** | **Feature Label** | **Mean AUPRC Decrease** | **SD** | **Evaluation Data** |
| --- | --- | --- | --- | --- |
| hw1 | Child age | 0.017741 | 0.004641 | Random sample of 12,000 held-out test observations |
| m5 | Breastfeeding category | 0.013155 | 0.001711 | Random sample of 12,000 held-out test observations |
| v106 | Maternal education | 0.011871 | 0.001587 | Random sample of 12,000 held-out test observations |
| m17 | Caesarean delivery | 0.008052 | 0.001673 | Random sample of 12,000 held-out test observations |
| v025 | Residence | 0.005432 | 0.000891 | Random sample of 12,000 held-out test observations |
| v717 | Respondent occupation | 0.003715 | 0.002112 | Random sample of 12,000 held-out test observations |
| v190 | Wealth index | 0.003122 | 0.001129 | Random sample of 12,000 held-out test observations |
| bmi | Child BMI category | 0.002354 | 0.001371 | Random sample of 12,000 held-out test observations |
| v705 | Father occupation | 0.002037 | 0.002056 | Random sample of 12,000 held-out test observations |
| b4 | Child sex | 0.000188 | 0.000143 | Random sample of 12,000 held-out test observations |
| v137 | Children aged <=5 | -6.72E-05 | 0.000509 | Random sample of 12,000 held-out test observations |
| h10 | Ever vaccinated | -0.00173 | 0.000716 | Random sample of 12,000 held-out test observations |

**Supplementary Table S10:** Complete candidate-model tuning results using common folds.

**Supplementary Table S10** can be found as a separate CSV file.
