# Supplementary material for: Comparison of Machine Learning Classifiers for Predicting Reported Antibiotic Treatment After Recent Fever/Cough Among Under‐Five Children: A Pooled Analysis of DHS Data From 64 LMICs
Source: Public Health Chall. 2026 Oct 9;5(4):e70413. doi: 10.1002/puh2.70413 (PMC13652348; doi:10.1002/puh2.70413)
Supplement: Supplementary file 2 [file PUH2-5-e70413-s006.docx]

**Supplementary Figure S1:** Precision–recall curves for all models on the held-out test set.

**Supplementary Figure S2:** Calibration plots by predicted-probability decile.
